# An intronic structure enabled by a long-distance interaction serves as a novel target for splicing correction in spinal muscular atrophy

**DOI:** 10.1093/nar/gkt609

**Published:** 2013-07-15

**Authors:** Natalia N. Singh, Mariah N. Lawler, Eric W. Ottesen, Daya Upreti, Jennifer R. Kaczynski, Ravindra N. Singh

**Affiliations:** ^1^Department of Biomedical Sciences, Iowa State University, Ames, IA 50011, USA, ^2^Department of Biochemistry, Iowa State University, Ames, IA 50011, USA, ^3^Molecular Cellular and Developmental Biology Program, Iowa State University, Ames, IA 50011, USA and ^4^Biology Program, Iowa State University, Ames, IA 50011, USA

## Abstract

Here, we report a long-distance interaction (LDI) as a critical regulator of alternative splicing of *Survival Motor Neuron 2* (*SMN2*) exon 7, skipping of which is linked to spinal muscular atrophy (SMA), a leading genetic disease of children and infants. We show that this LDI is linked to a unique intra-intronic structure that we term internal stem through LDI-1 (ISTL1). We used site-specific mutations and Selective 2′-Hydroxyl Acylation analyzed by Primer Extension to confirm the formation and functional significance of ISTL1. We demonstrate that the inhibitory effect of ISTL1 is independent of hnRNP A1/A2B1 and PTB1 previously implicated in *SMN2* exon 7 splicing. We show that an antisense oligonucleotide-mediated sequestration of the 3′ strand of ISTL1 fully corrects *SMN2* exon 7 splicing and restores high levels of SMN and Gemin2, a SMN-interacting protein, in SMA patient cells. Our results also reveal that the 3′ strand of ISTL1 and upstream sequences constitute an inhibitory region that we term intronic splicing silencer N2 (ISS-N2). This is the first report to demonstrate a critical role of a structure-associated LDI in splicing regulation of an essential gene linked to a genetic disease. Our findings expand the repertoire of potential targets for an antisense oligonucleotide-mediated therapy of SMA.

## INTRODUCTION

Alternative splicing is modulated by combinatorial control exerted by overlapping linear motifs called exonic or intronic splicing enhancers and silencers (ISSs) (1–3). Although methods to define linear splicing motifs continue to evolve (4–6), there is a growing appreciation of the role of RNA structure in regulation of alternative splicing (7–10). RNA secondary structure folding occurs on a microsecond time scale (11,12), a rate that is faster than polymerase II-mediated transcription elongation, which is ∼100 nt per second (13). Therefore, terminal stem-loops (TSLs), which represent the most prevalent form of local structures, are formed as soon as the nascent transcript emerges from the polymerase. Multiple studies confirm the role of TSLs in modulation of alternative splicing (14–18). Evidence suggests that internal stems formed by long-range interactions affect pre-mRNA splicing as well (8,19,20). However, functional validation of such interactions as critical checkpoints for splicing regulation in the context of a human disease has not been done.

**Figure 1.**
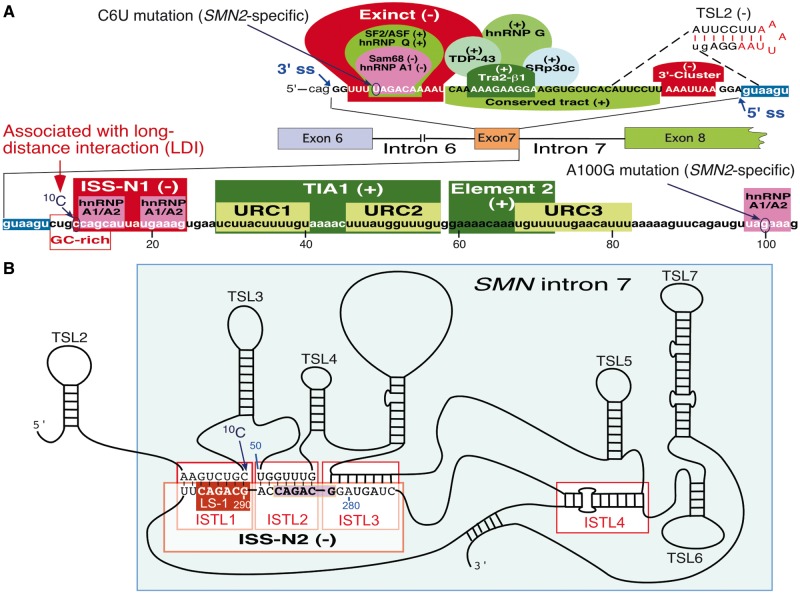
An account of transacting factors and cis-elements including RNA secondary structure that regulate *SMN2* exon 7 splicing. (**A**) Diagrammatic representation of *cis-*elements located within exon 7 and the first 104 nt of intron 7 of *SMN2*. The sequence of *SMN2* exon 7 and adjacent intron 7 are given. Numbering of nucleotides starts from the beginning of intron 7. Positive *cis*-elements/transacting factors that promote exon 7 inclusion and negative *cis*-elements/transacting factors that promote exon 7 skipping are indicated by (+) and (−), respectively. Exinct, Conserved tract and 3′-Cluster were identified by *in vivo* selection of the entire exon 7 (26). TSL2 structure sequesters the 5′ ss of exon 7 (16). Element 2 and binding sites for SF2/ASF, hnRNP A1/A2, Sam68, hnRNP Q, Tra2-β1, TDP-43, hnRNP G and SRp30c were described by others (27). TIA1 was shown to bind to intron 7 U-rich Clusters (URCs) 1 and 2 and promote exon 7 inclusion (28). ISS-N1, along with an overlapping GC-rich sequence and the ^10^C involved in LDI all contribute toward exon 7 skipping (27). (**B**) Schematic representation of RNA secondary structure of *SMN2* intron 7. The schematic is based on chemical structure probing performed in this study (see Figure 6). A defining feature of the RNA secondary structure of *SMN2* intron 7 is the presence of the three adjacent internal stems formed by LDIs (ISTLs). The adjacent 3′-strands of ISTL1, ISTL2 and ISTL3 constitute ISS-N2, a novel target for splicing correction in SMA (described later). Of note, ^10^C is locked in ISTL1 and base pairs with the 290th position of *SMN2* intron 7. A sequence identical to LS-1 has been shaded. Descriptions of abbreviations are given in Supplementary Table S1.

Humans have two nearly identical copies of the *Survival Motor Neuron* (*SMN*) gene: *SMN1* and *SMN2* (21). The two *SMN* genes code for identical proteins; however, *SMN2* predominantly generates a shorter transcript owing to skipping of exon 7, which produces a truncated, unstable SMN (22,23). The inability of *SMN2* to compensate for the loss of *SMN1* results in spinal muscular atrophy (SMA), a debilitating childhood disease (24). *SMN2* exon 7 skipping is caused by a C-to-T mutation at the sixth position (C6U in transcript) of exon 7 (25). C6U weakens the 3′ splice site (3′ ss) owing to the loss of an exonic splicing enhancer associated with SF2/ASF and/or gain of an exonic splicing silencer associated with hnRNP A1 [Figure 1, (29,30)]. Another *SMN2*-specific mutation at the 100th position of intron 7 creates an ISS associated with hnRNP A1 [Figure 1, (31)]. Sam68 and PTB1 are two additional inhibitory proteins implicated in *SMN2* exon 7 skipping [Figure 1, (28,32,33)]. Several positive factors, including hnRNP G, hnRNP Q, SRp30c, TDP43, TIA1 and Tra2-β1 stimulate *SMN2* exon 7 inclusion [Figure 1, (27)].

An early *in vivo* selection study to unravel the position-specific role of residues within exon 7 revealed the suboptimal nature of its 5′ ss (26). Subsequent studies uncovered a series of negative *cis*-elements in the vicinity of the 5′ ss of exon 7, including ISS-N1, a stem-loop structure (TSL2) and a GC-rich sequence that partially overlaps with ISS-N1 [Figure 1, (16,34,35)]. The discovery of ISS-N1 was particularly significant, as it served as the first example in which deletion of an inhibitory intronic element fully restored *SMN2* exon 7 inclusion even in the absence of the critical positive regulatory elements within exon 7 (34). Further, sequestration of ISS-N1 by an antisense oligonucleotide (ASO) corrected *SMN2* exon 7 splicing and restored high levels of SMN protein in SMA patient cells. Of note, different mechanisms may account for the strong stimulatory effect of ISS-N1 deletion and ASO-mediated ISS-N1 sequestration. For example, deletion of ISS-N1 brings a TIA1-binding site (a positive *cis*-element) close to the 5′ ss of exon 7 (28), whereas an ASO-mediated sequestration of ISS-N1 destabilizes an inhibitory structure close to the 5′ ss of exon 7 (this study). Following the discovery of ISS-N1 in 2006, an unprecedented number of *in vivo* studies independently confirmed the therapeutic efficacy of ISS-N1-targeting ASOs (36–39).

We have previously reported a unique long-distance interaction (LDI) between a cytosine residue at the 10th position (^10^C) of *SMN2* intron 7 and a downstream intronic sequence separated from ^10^C by hundreds of nucleotides (40). The most surprising aspect of this discovery was the position-specific role of ^10^C. An ASO (F14) that sequestered the first 14 residues (including ^10^C) of ISS-N1 promoted *SMN2* exon 7 inclusion, whereas another ASO (L14) that sequestered the last 14 residues (excluding ^10^C) of ISS-N1 increased *SMN2* exon 7 skipping. Deletion or substitution of ^10^C fully abrogated the inhibitory effect of L14, confirming that the negative impact of this ASO is associated exclusively with the unsequestered ^10^C. The first indirect support linking ^10^C to an LDI came from experiments in a heterologous system, where the negative effect of L14 on exon 7 splicing was not recapitulated, despite the system harboring the entire *SMN2* exon 7 and flanking intronic sequences, including ISS-N1 (40). Subsequent experiments revealed that ^10^C engages in a LDI with downstream sequences within the 3′ portion of *SMN2* intron 7. Indeed, deletion of these sequences transformed L14 into a stimulatory ASO (40). These findings offered a unique opportunity to uncover the molecular basis of a rare LDI in the context of a leading genetic disease.

Here, we report the mechanistic basis of the ^10^C-mediated LDI, which we found to be facilitated by an intricate arrangement of sequence and structural motifs within *SMN2* intron 7. Using deletions and site-specific mutations combined with chemical structure probing, we demonstrate that the ^10^C-mediated LDI is linked to a unique RNA structure. We also show that the inhibitory effect of the ^10^C-mediated LDI is independent of the widely expressed hnRNP A1/A2B1, and PTB1 proteins that were previously implicated in skipping of *SMN2* exon 7. These results provide the first example in which a deep intronic sequence associated with a RNA structure supported by a unique LDI modulates alternative splicing in a major genetic disease. In addition, we demonstrate that a RNA structure within an intron could serve as an effective target for an ASO-mediated splicing correction in SMA.

## MATERIALS AND METHODS

### Minigenes and antisense oligonucleotides

*SMN2* mutant minigenes were generated by PCR using a strategy described earlier (26). The identity of the newly constructed minigenes was verified by sequencing. All primers for cloning were from Integrated DNA Technologies. Reagents for PCR and cloning were from New England Biolabs. RNA ASOs were synthesized by Dharmacon Inc. and incorporated a phosphorothioate backbone and 2′-O-methyl modifications at each base [Supplementary Table S2, (34)].

### Cell culture

All tissue culture media and supplies were purchased from Life Technologies. Human cervical adenocarcinoma (HeLa) cells obtained from the American Type Culture Collection were cultured in Dulbecco’s modified Eagle’s medium supplemented with 10% fetal bovine serum. Primary patient fibroblasts from an SMA type I patient (repository number GM03813) were obtained from Coriell Cell Repositories. These cells were grown in minimal essential medium (MEM, catalog # 10370) supplemented with 1X GlutaMAX-I and 15% fetal bovine serum.

### Cell transfection

Transient transfections of cells with plasmid DNA and/or ASOs were performed using Lipofectamine-2000 (Life Technologies) following the manufacturer’s recommendations. HeLa cells were plated at a density of ∼1.1 × 10^5^ cells per well of 24-well plates. The next day, cells were cotransfected with 0.1 µg of a given minigene and 50 nM of an ASO of interest. The total amount of transfected nucleic acid was maintained constant (0.8 µg) by adding the control ASO [Supplementary Table S2, (40)]. The growth medium was changed 6 h post-transfection. In the case of primary fibroblasts, depending on the amount of cells needed for the analysis, GM03813 cells were plated in either 6-well plates or 100 mm dishes at a density of 1.3 × 10^5^ cells per well or 9 × 10^5^ cells per dish, respectively. The next day, GM03813 cells were transfected with ASOs. ASO concentrations varied and are indicated in the legend of Figure 11. The growth medium was replaced 6 h after transfection. For isolation of total RNA only, HeLa and GM03813 cells were lysed directly in tissue culture plates ∼24 h post-transfection using Trizol Reagent (Life Technologies). For simultaneous RNA and protein analysis, transfected GM03813 cells were collected 48 h post-transfection by scraping. One-third of the cells were taken for RNA isolation with Trizol, and the rest were used for protein isolation and western blotting.

### ASO transfection combined with protein knockdown

Proteins of interest were knocked down using siRNAs (ON-TARGETplus SMART pool; Dharmacon Inc.). An ON-TARGETplus non-targeting pool served as a control siRNA. To knockdown proteins of interest, HeLa cells were reverse transfected with siRNAs twice with an interval of ∼48 h. For each siRNA transfection, a siRNA-Lipofectamine-2000 complex was prepared following the manufacturer’s suggestions, combined with HeLa cell suspension containing ∼1 × 10^6^ cells in a total volume of 2 ml, and then the cells were seeded in one well of a 6-well plate. For simultaneous transfection of siRNAs against hnRNP A1 and hnRNP A2B1, two wells were seeded, as simultaneous depletion of hnRNP A1 and hnRNP A2B1 proteins had a negative effect on cell survival. For the first transfection, the final concentration for individual siRNAs was 80 nM and 40 nM each when siRNAs against hnRNP A1 and hnRNP A2B1 were transfected together. The next day, the transfected cells were trypsinized and transfered to 60 mm dishes. The second reverse transfection of HeLa cells with siRNAs was performed ∼48 h after the first one essentially as described earlier in the text, except that the siRNA concentration was increased to 100 nM for individual siRNAs and to 50 nM each when siRNAs against hnRNP A1 and hnRNP A2B1 were transfected together. Twenty-four hours after the second siRNA transfection, HeLa cells were trypsinized and seeded in 24-well plates to be transfected with the ASOs of interest the next day. The remaining cells were returned to 6-well plates to be collected ∼22 h later for making cell lysates to monitor the efficiency of protein knockdown. Three wells of a 24-well plate were seeded for each siRNA transfection. The density of plating per well was ∼1.4 × 10^5^ cells for HeLa transfected with a control siRNA, ∼1.6 × 10^5^ cells for HeLa transfected with individual siRNA against hnRNP A1, hnRNP A2B1 or PTB1 and ∼1.7 × 10^5^ cells for HeLa co-transfected with siRNAs against hnRNP A1 and hnRNP A2B1. HeLa cells seeded in 24-well plates were transfected ∼24 h later with 100 nM ASO of interest along with corresponding siRNA using Lipofectamine-2000 followed by cell lysis ∼24 hours later and isolation of total RNA.

### *In vivo* splicing assay

Total RNA was isolated using Trizol Reagent following the manufacturer’s instructions. cDNA was generated as described previously (40). Minigene-specific spliced products were amplified using Taq DNA polymerase and the P1 and P2 primer pair [Supplementary Table S3, (34)]. For PCR amplification of endogenous *SMN*, either P25 and P31 or N-24 and P2 primer pairs were used [Supplementary Table S3, (28,34)]. Cloning and sequencing confirmed the identity of splice variants amplified by RT-PCR (26). To accurately determine the relative abundance of splice variants, reduced cycles of PCR reactions were performed either in the presence of a trace amount of [α-^32^P] dATP (3000 Ci/mmole, Perkin-Elmer Life Sciences) or with the P2 primer labeled at the 5′-end with ^32^P. To distinguish *SMN1* and *SMN2* splice isoforms, PCR products amplified with primers P25 and P31 were subjected to overnight DdeI digestion, followed by phenol:chloroform extraction and ethanol precipitation (28). Quantification and analysis of splice products were performed using an FPL-5000 Image Reader and Multi Gauge software (Fuji Photo Film Inc.). Results were confirmed by at least three independent experiments. The percentage values of exon 7 skipping given in figures were calculated from the shown representative gel.

### Western blot analysis

HeLa cells (∼3 × 10^5^) were harvested, and cell lysates were prepared similarly as described before (34). One-seventh of each lysate was used for one blot. Whole-cell extracts from GM03813 cells were prepared as described previously (41). Protein concentrations were determined using the Bio-Rad Protein Assay Dye Reagent Concentrate (Bio-Rad). Protein samples were resolved on 8 or 10% SDS–polyacrylamide gels and transferred onto polyvinylidene fluoride membrane (Bio Trace PVDF, Pall Life Sciences). The following primary and secondary antibodies were used for western blot analysis: mouse monoclonal anti-PTB1 (Abcam), mouse monoclonal anti-hnRNP A1, clone 9H10 (Abcam), mouse monoclonal anti-hnRNP A2B1, clone DP3B3 (Abcam), mouse monoclonal anti-SMN, clone 8 (BD Transduction Laboratories), mouse monoclonal anti-Gemin2, clone 2E17 (Sigma), mouse monoclonal anti-GAPDH, clone 6C5 (Abcam), rabbit polyclonal anti-Actin (Sigma-Aldrich), goat anti-mouse horseradish peroxidase-conjugated antibody (Jackson Immunoresearch) and donkey anti-rabbit horseradish peroxidase-conjugated antibody (GE Healthcare). Immunoreactive proteins were visualized with Clarity Western ECL substrate (Bio-Rad), SuperSignal West Dura Extended Duration Substrate or SuperSignal West Femto Maximum Sensitivity Substrate (Thermo Scientific). Membranes were stripped using Restore Western Blot Stripping Buffer (Thermo Scientific) and re-probed for proteins of interest. The membranes were scanned using the UVP BioSpectrum AC Imaging System (UVP). Results were confirmed by at least three independent experiments.

### RNA structure probing

RNA secondary structure was probed using Selective 2′-Hydroxyl Acylation analyzed by Primer Extension (SHAPE) following recommendations provided in (42). The 1-methyl-7-nitroisatoic anhydride (1M7) was synthesized as described (43). RNA substrates for structure probing were in vitro transcribed using T7 MEGAshortscript kit (Ambion) following the manufacturer’s recommendations. Templates for T7 *in vitro* transcription were prepared as follows. Sequences that contained the last 17 nt of exon 7 and all of intron 7 were amplified by PCR using the primer pair 5′T7-Xba-2 (5′-ATA TAT TCT AGA TAA TAC GAC TCA CTA TAG GGA TTC CTT AAA TTA AGG AGT AAG TC-3′) and 3′Hind-2 (5′-ATA TAT AAG CTT TTC TGC AAA TGA GAA ATT AGA ACC AG-3′) and either wild-type SMN2ΔI6 minigene (26) or internal stem through LDI-1 (ISTL1)-M4 mutant (this study) as a template. The resulting PCR fragments were digested with XbaI and HindIII and cloned into the pUC19 vector to generate the plasmid constructs T7-TSL2-In7 and T7-TSL2-ISTL1-M4. The plasmids were linearized with HindIII and used as tempates for RNAs synthesized in an overnight T7 transcription reaction. The DNA template was removed by DNase treatment followed by phenol:chloroform extraction, and RNA was recovered by ethanol precipitation, dissolved in water and further purified by centrifugation through a gel-filtration Micro Bio-spin P-30 Chromatography column (Bio-Rad).

Primers used for extension reactions are listed in Supplementary Table S4. Extension primers (60 pmol each) were 5′-end-labeled using [γ-^32^P]ATP (6000 Ci/mmol) and T4 polynucleotide kinase (New England Biolabs), phenol:chloroform extracted, and centrifuged through a gel-filtration Micro Bio-spin P-30 Chromatography column to remove unincorporated [γ-^32^P]ATP. The eluted sample was evaporated under vacuum to reduce the volume to ∼5 µl to which 10 µl of formamide gel-loading buffer (Ambion) was added. End-labeled primers were then purified on denaturing 15% polyacrylamide gels by the ‘crush and soak’ method (16). The elution was done overnight at 37°C. Primers were ethanol precipitated, dissolved in 70 µl of water and centrifuged through a Micro Bio-spin P-30 Chromatography column. Of note, 1 µl of recovered primer was used for each 5 µl of primer extension reaction.

Before structure probing RNA was refolded as follows. In all, 16 pmol RNA with the control ASO (40) or ASO of interest was heated to 95°C for 2 min in 30 mM Tris–HCl (pH 8.0) and snap-cooled on ice. The amount of ASO used was 160 pmol for F14, L14 and ASO-M. The amount was increased to 204 pmol for ASO-D. Folding was performed in a total volume of 72 µl [100 mM Tris–HCl (pH 8.0), 100 mM NaCl and 10 mM MgCl_2_] at 37°C for 20 min after which time, the reaction was split into two aliquots (36 µl each), and the incubation continued for another 10 min. Four microliters of either 1M7 (100 mM in DMSO) or DMSO (control) were then added to the 36 µl of RNA folding reaction, and the mixture was incubated at 37°C for 2 min. Each modification reaction was then transferred to a tube containing 158 µl of water, 20 µl of potassium acetate [3 M (pH 5.5)], 0.8 µl of 500 mM EDTA (pH 8.0) and 1.2 µl of GlycoBlue (15 mg/ml, Ambion). RNA was recovered by ethanol precipitation and dissolved in 10 µl of water. The 1M7 modification sites were analyzed by primer extension using SuperScript III RTase (42). Briefly, modified RNA (1 µl) was mixed with 5′-end-labeled primer of interest (1 µl), 1 µl of water, 0.25 µl of 5X RT buffer and 0.25 µl of dNTP mixture (10 mM each). The mixture was heated to 65°C for 5 min and snap-cooled on ice. One microliter of RT mix (four parts 5X RT buffer and one part 0.1 M DTT) and 0.5 µl of SuperScript III RTase (100 U/µl) were then added to the primer extension reaction. The reaction was incubated for 2 min at 45°C and 30 min at 55°C. To prevent RTase falloffs at positions 292 and 293 routinely observed with extension primer#17 and primer#315, ASO-D (5 pmol) was added to the reaction mixture before the denaturation step. Denaturation itself was performed at 95°C for 2 min instead of at 65°C for 5 min. Also, before RTase addition, the reaction was incubated for 5 min at 45°C to allow ASO-D to invade the ISTL1 structure and release its 3′ strand for primer extension. To generate sequencing ladders, primer extension reactions were performed as described earlier in the text, except that 0.5 µl of a given ddNTP (10 mM) was added per 5 µl of reaction and 40 ng of the wild-type RNA substrate was used as a template. For the primer extension step, the reactions were incubated for 2 min at 45°C and 15 min at 55°C. To degrade RNA after completion of primer extension, 0.5 µl of 2 M NaOH was added to the reaction, and the mixture was heated at 95°C for 5 min. The mixture was then allowed to cool to room temperature, and 1 µl of 1 M unbuffered Tris–HCl solution was added to neutralize the pH. The products of the primer extension reaction were resolved on denaturing 6% polyacrylamide gels and visualized by autoradiography using a FPL-5000 Image Reader.

Band intensities were quantified using Multi Gauge software (Fuji Photo Film Inc). First, 1M7 reactivity for each position was calculated by subtracting the control (DMSO) band intensity from the ‘1M7-modified’ band intensity. For each extension primer, the reactivity of nucleotides was then normalized relative to the most reactive position, which was assigned a value of 1. Nucleotides with normalized reactivity between 0.3 and 0.5 were considered to be moderately reactive with 1M7, whereas nucleotides with normalized reactivity above 0.5 were considered to be highly reactive with 1M7.

## RESULTS

### Characterization of intronic sequences involved in LDI

**Figure 2.**
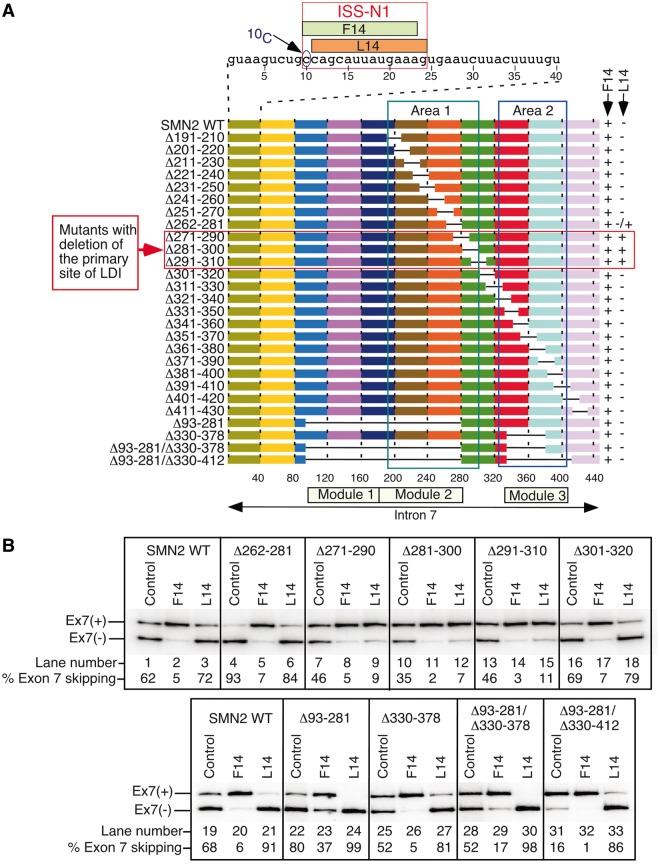
Identification of the primary site of the ^10^C-mediated LDI. (**A**) Diagrammatic representation of 20 nt long overlapping deletions within *SMN2* intron 7. The sequence of the first 40 nucleotides of intron 7 is given at the top, with F14 and L14 target sites within ISS-N1 indicated. Deletions are shown as lines. Mutants’ names are given on the left. Numbers in mutants’ names represent the positions at which deletions were made. The effect of F14 and L14 on splicing is indicated on the right with (+) (promotes exon 7 inclusion) and (−) (promotes exon 7 skipping). (**B**) *In vivo* splicing pattern of the wild-type *SMN2* minigene and the representative deletion mutants from panel (A) in the presence of the indicated ASO. Exon 7-included (+) and exon 7-skipped (−) spliced products are marked. Control represents transfection with 10mer ASO (Supplementary Table S2). Results were analyzed as described in (26). Ex7, exon 7.

We previously reported that the ^10^C-mediated LDI is modulated by sequences from the 195th to 306th (Area 1) and 325th to 405th (Area 2) positions of *SMN2* intron 7 [Figure 2A, (40)]. To understand the mechanistic basis of the ^10^C-mediated LDI, we thought it essential to link the L14-induced inhibitory effect of ^10^C to the smallest possible sequence. For this, we generated a set of 23 *SMN2* minigenes carrying 20 nt long overlapping deletion mutations encompassing Area 1 and Area 2 (Figure 2A). We then co-transfected HeLa cells with these minigenes and F14 or L14 and determined the splicing pattern of exon 7 at ∼24 h post-transfection. As shown in Figure 2B, L14 increased exon 7 skipping in the majority of mutants except Δ271–290, Δ281–300 and Δ291–310, and to a lesser extent Δ262–281, whereas F14 caused predominant exon 7 inclusion in all cases. These results refined the region of the LDI as a single sequence stretch between positions 271 and 300 within Area 1; we consider it to be the primary site of LDI. Consistently, L14 retained the inhibitory effect on exon 7 splicing in *SMN2* minigenes carrying large deletions outside of the identified site, namely, spanning the region from position 93 to 281 and position 330 to 412 (Figure 2B, lanes 22–33). Of note, these large deleted sequences fold into independent secondary structures or modules (Modules 1, 2 and 3; Figure 2A) as determined by structure probing (described later). To summarize, our results of large deletions and the 20 nt long overlapping deletions narrowed the primary site of the ^10^C-mediated LDI to an area between the 282nd and 300th positions of intron 7.

**Figure 3.**
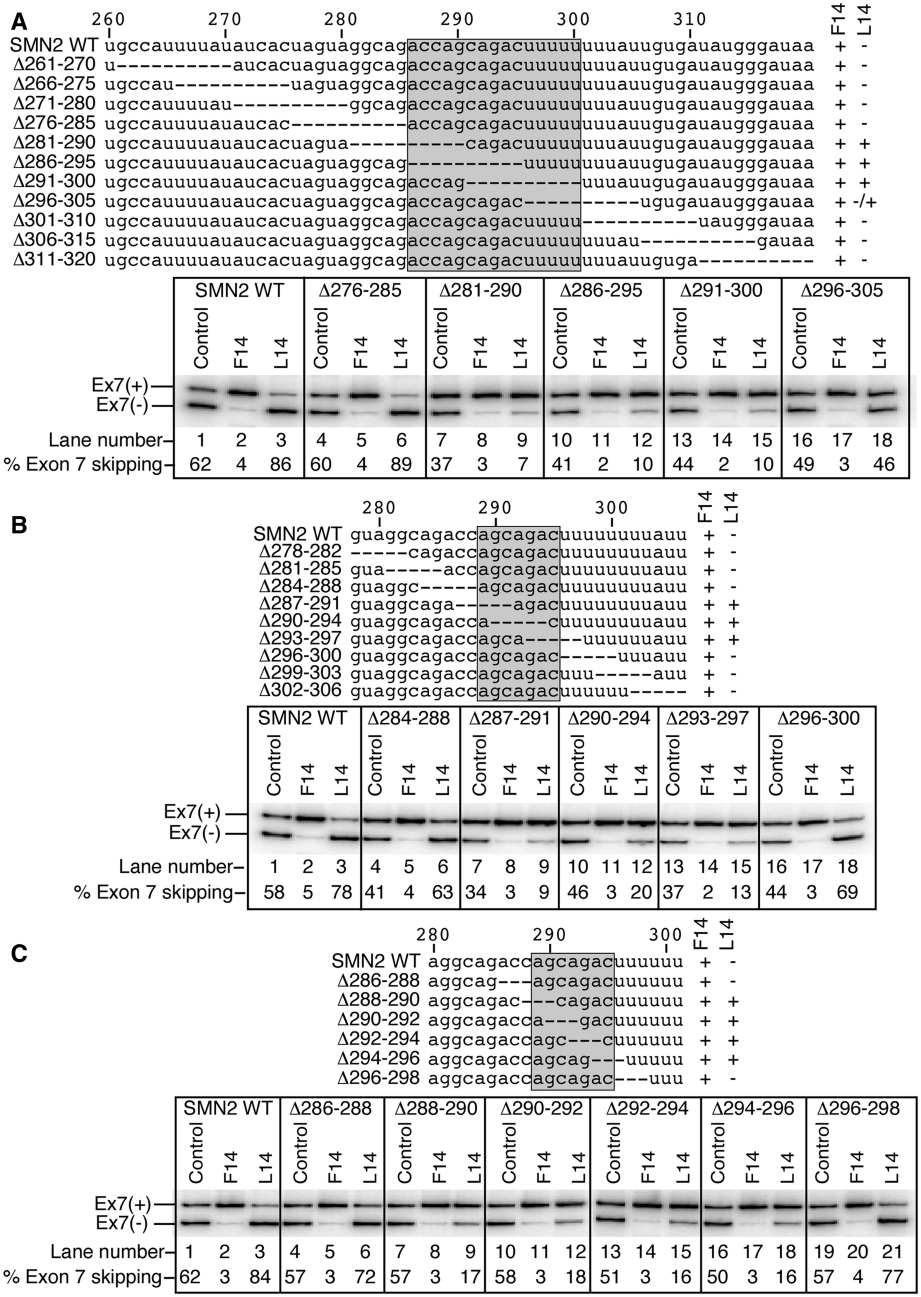
Characterization of the smallest motif of the ^10^C-mediated LDI. Intron 7 sequences of 10- (**A**), 5- (**B**) and 3 nt long (**C**) overlapping deletion mutants are shown with deletions indicated as dashed lines. Nucleotide numbering starts from the beginning of intron 7. Mutants’ names are given on the left. Numbers in mutants’ names represent the positions that were deleted. The effect of F14 and L14 on splicing is indicated similarly as in Figure 2. The region determined to be an interacting partner of ^10^C is highlighted. *In vivo* splicing patterns of the wild-type *SMN2* minigene and the representative deletion mutants in the presence of the indicated ASOs are given in the bottom panels. Control ASO was the same as described in Figure 2. Results were analyzed as described in (26).

To further define the sequence motif that interacts with ^10^C, we examined the splicing pattern of *SMN2* mutants carrying 10 nt long overlapping deletions from the 261st to 320th positions of intron 7 (Figure 3A, upper panel). As expected, F14 effectively stimulated exon 7 inclusion in all 11 mutants examined, whereas L14 inhibited exon 7 inclusion in all but four mutants (Δ281–290, Δ286–295, Δ291–300, and to a lesser extent Δ296–305) (Figure 3A, lower panel). These results further narrowed the primary site of the ^10^C-mediated LDI to a single stretch of 15 nt from the 286th to 300th positions of intron 7 (Figure 3A, upper panel). Also, underscoring the inhibitory nature of this sequence, the 10 nt long overlapping deletions within this region themselves produced a noticeable stimulatory effect on *SMN2* exon 7 splicing (Figure 3A, lanes 1, 7, 10, 13 and 16).

To continue our effort to find the smallest possible motif that is engaged in the LDI with ^10^C, we constructed a set of mutants carrying 5 nt long overlapping deletions within the sequence spanning from the 284th to 300th positions of *SMN2* intron 7 (Figure 3B, upper panel) and examined their splicing pattern in the presence of F14 or L14. We identified three overlapping deletion mutants (Δ287–291, Δ290–294 and Δ293–297) in which the ability of L14 to increase skipping of exon 7 was lost (Figure 3B, lower panel). Thus, the primary site of the LDI was reduced to a single 7 nt long sequence, AGCAGAC, from the 289th to 295th positions of intron 7 (Figure 3B, upper panel). We also performed a parallel experiment using *SMN2* mutant minigenes that carried 3 nt long overlapping deletions within the sequence spanning from the 286th to 298th positions of intron 7 (Figure 3C, upper panel). The results of this experiment identified the same 7 nt long motif, AGCAGAC, from position 289 to position 295 as the core site of the ^10^C-mediated LDI (Figure 3C).

### The site of LDI maps to a unique RNA secondary structure

**Figure 4.**
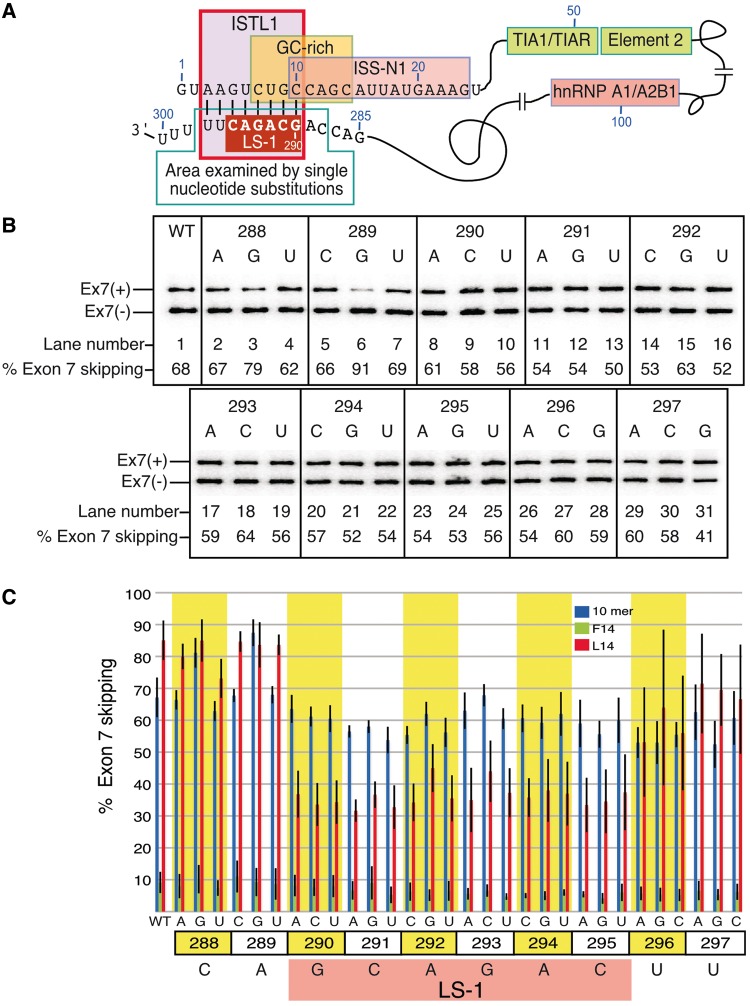
Effect of point mutations within the smallest motif associated with the ^10^C-mediated LDI. (**A**) Diagrammatic representation of regulatory *cis*-elements/transacting factors and their relative arrangement in the context of *SMN2* intron 7. The sequences of the first 25 nt and from the 285th to 300th positions of intron 7 are given. The region targeted for single-nucleotide substitutions is highlighted. (**B**) *In vivo* splicing pattern of *SMN2* mutants with single-nucleotide substitutions. Numbers and letters at the top of the gel represent the positions and the type of substitutions within intron 7. Spliced products are the same as those indicated in Figure 2B. Results were analyzed as described in (26). (**C**) Bar diagram showing the percentage of exon 7 skipped in the presence of the control ASO (blue), F14 (green) and L14 (red). Control ASO was the same as described in Figure 2. Error bars represent standard deviations (minimum of three replicates). Numbers and letters at the bottom of the bar diagram represent the positions and the type of substitutions within intron 7. Every non-wild-type nucleotide substitution within the highlighted LS-1 abrogated the negative effect of L14.

To assess the position-specific impact of residues involved in the interaction with ^10^C, we examined the effect of point mutations in a 10 nt long sequence stretch between intronic positions 288 and 297 on splicing of *SMN2* exon 7 (Figure 4). This sequence contains the newly identified site of the ^10^C-mediated LDI with a few upstream and downstream residues. Interestingly, all but 1 nt of this sequence also constitute the 3′ strand of a *mfold* (44) predicted secondary structure that we call internal stem through LDI-1 (ISTL1) (Figure 4A). The 5′ strand of ISTL1 incorporates ^10^C. Consistent with the anticipated inhibitory role of ISTL1, a number of point mutations within the 3′ strand of ISTL1 caused an improvement in *SMN2* exon 7 inclusion (Figure 4B). At the same time, an A to G substitution at the 289th position, which is predicted to extend the size of the ISTL1 duplex by 1 bp, produced a strong inhibitory effect on *SMN2* exon 7 splicing (Figure 4B, lane 6). This finding further underscored the inhibitory role of ISTL1.

We next examined the effect of F14 and L14 on splicing of exon 7 in *SMN2* minigenes carrying point mutations described in Figure 4B. L14 produced a stimulatory effect on exon 7 splicing in all *SMN2* minigenes with point mutations in the sequence spanning the 290th–295th positions of *SMN2* intron 7 (Figure 4C). Therefore, we conclude that this sequence (GCAGAC) constitutes the LDI partner of ^10^C. To emphasize the sequence-specific and position-dependent nature of the ^10^C-mediated LDI, we term the GCAGAC motif spanning the 290th–295th positions of intron 7 as LDI Site-1 or LS-1 (Figure 4C). As all six residues of LS-1 fall within the 3′ strand of ISTL1 (Figure 4A), we attribute the stimulatory effect of L14 on exon 7 splicing in LS-1 mutants to the destabilization of ISTL1. Remarkably, deletion of LS-1 but not an identical upstream GCAGAC motif abrogated the ^10^C-mediated LDI (Supplementary Figure S1). These results underlined the requirement of a specific sequence motif at a precise location within intron 7 for the interaction with ^10^C. These results are also consistent with a role of RNA structure in which specific positioning of LS-1 in the context of ISTL1 facilitates the LDI.

### Role of ISTL1 in regulation of *SMN2* exon 7 splicing

**Figure 5.**
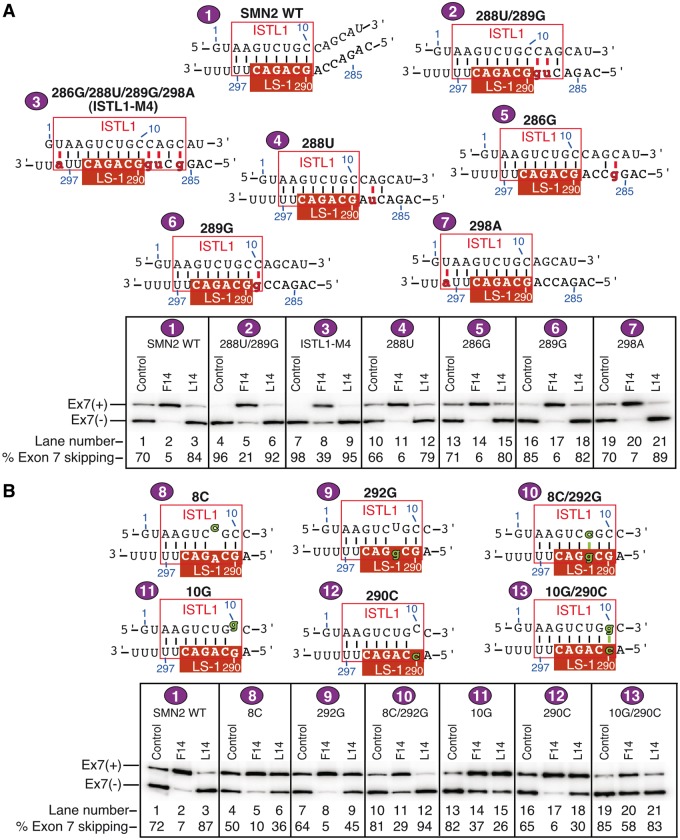
Effect of structure-associated mutations on the ^10^C-mediated LDI. (**A**) Upper panel shows diagrammatic representation of predicted ISTL1 strengthened by mutations indicated in lower-case letters and highlighted in red. New base pairs formed owing to these mutations are shown as red lines. Each minigene was assigned a name and a number as shown at the top of each structure. *In vivo* splicing patterns of minigenes in the presence of the indicated ASOs are shown in the bottom panel. Spliced products are the same as those marked in Figure 2B. Control ASO was the same as described in Figure 2. Results were analyzed as described in (26). (**B**) Upper panel shows diagrammatic representation of the predicted ISTL1 destabilized or restored by mutations indicated in lower-case letters and highlighted in green. Restored base pairs formed due to the mutations are shown as green lines. Other descriptions are same as in panel A.

To evaluate the impact of the ISTL1 structure on exon 7 splicing, we generated an *SMN2* minigene with a double mutation (288U/289G), which is predicted to extend the length of ISTL1 by 3 bp. This mutant showed greatly increased exon 7 skipping as compared with the wild-type construct (Figure 5A, lane 4). Furthermore, another minigene mutant, ISTL1-M4, which carried four substitutions (286G/288U/289G/298A), and therefore is predicted to have at least a 13 bp long ISTL1, showed near-total skipping of exon 7 (Figure 5A, lane 7), confirming that the inhibitory effect of ISTL1 is proportional to the size of the stem. In both mutants, the target site of F14 is partially sequestered by ISTL1. Consequently, in both mutants, F14 had a diminished stimulatory effect on exon 7 splicing (Figure 5A, lanes 5 and 8). We also compared the effect of single nucleotide substitutions at the 286th, 288th, 289th and 298th positions of intron 7. Although both 289G and 298A mutants are predicted to extend ISTL1 by 1 bp, only 289G showed a noticeable increase in *SMN2* exon 7 skipping (Figure 5A, lanes 16 and 19). This difference could be due to the fact that the A:U base pair formed by 298A is weaker than the G:C base pair formed by 289G (Figure 5A, upper panel). As expected, 288U and 286G mutations that did not increase the size of ISTL1 had no appreciable gain in the inhibitory effect on *SMN2* exon 7 splicing (Figure 5A, lanes 10 and 13).

To functionally validate the role of ISTL1 on *SMN2* exon 7 splicing, we tested the effect of compensatory mutations. In particular, we substituted U for C at the 8th (8C) and C for G at the 10th (10G) positions of *SMN2* intron 7. The 8C and 10G mutants had stimulatory and inhibitory effects on exon 7 splicing, respectively (Figure 5B, lanes 4 and 13). The unexpected negative effect of the 10G mutation could be due to creation and/or strengthening of an existing inhibitory element. Similar to point mutations within the 3′ strand of ISTL1 (Figure 4), these mutations are predicted to destabilize ISTL1. Consistently, L14 lost its inhibitory effect and promoted inclusion of exon 7 in both 8C and 10G mutants (Figure 5B, lanes 6 and 15). These results further supported the hypothesis that destabilization of ISTL1 is necessary to abrogate the ^10^C-mediated LDI. In the context of the wild-type *SMN2* intron 7, 8U and 10C residues are predicted to base pair with 292A and 290G, respectively. Mutations at either the 290th or 292nd positions abrogated the negative effect of L14 (Figure 4C; Figure 5B, lanes 9 and 18). We predicted that in the minigenes 8C/292G and 10G/290C that carry restored ISTL1, L14 would either regain its negative effect on exon 7 splicing or suppress the stimulatory effect it produced in minigenes carrying single nucleotide mutations. Indeed, L14 noticeably increased exon 7 skipping in the 8C/292G mutant and suppressed the stimulatory effect in case of 10G/290C (Figure 5B, lanes 12 and 21). These results provided strong evidence in support of the modulatory role of ISTL1 structure in *SMN2* exon 7 splicing.

### Structure probing supports ISTL1 formation

**Figure 6.**
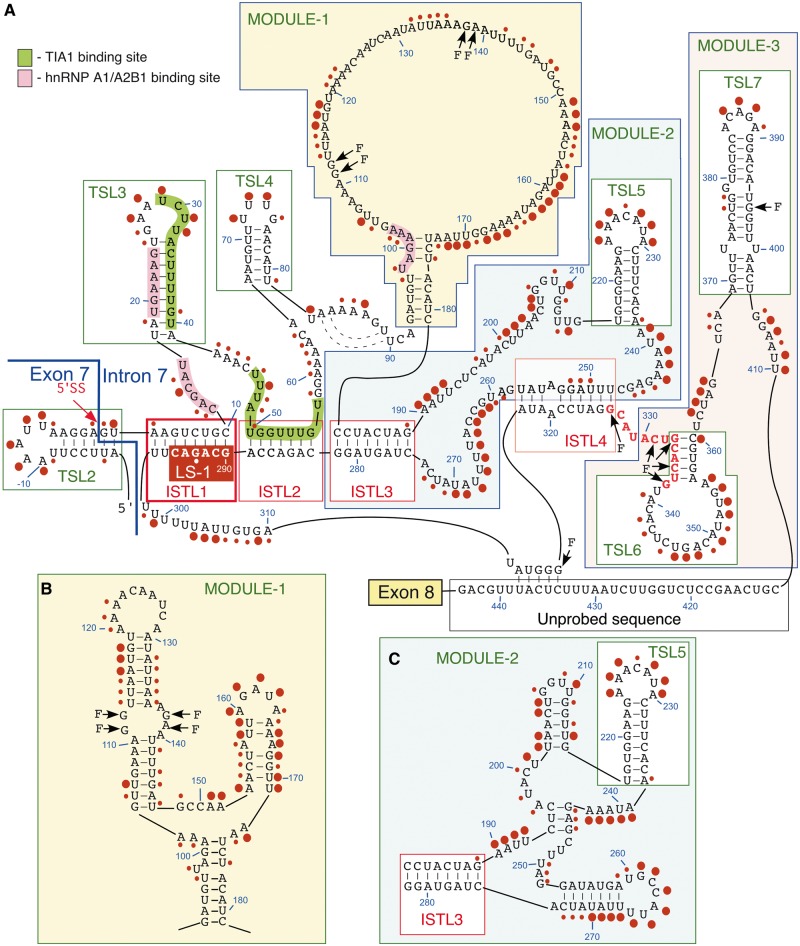
Secondary structure of *SMN2* intron 7. (**A**) SHAPE-derived structure of *SMN2* intron 7. This structure is based on combined results produced with 10 extension primers (Supplementary Table S4). Large circles indicate nucleotides with normalized 1M7 reactivity >0.5, small circles indicate nucleotides with normalized 1M7 reactivity between 0.3 and 0.5. Locations of modules, ISTLs and TSLs have been indicated. Positions corresponding to RTase falloffs are marked with ‘F’. Nucleotides marked in red constitute a region with unconfirmed structure owing to multiple falloffs. Alternative secondary structures of Module 1 (**B**) and Module 2 (**C**) as predicted by *mfold*. Descriptions of abbreviations are given in the main body of the text as well as in the Supplementary Table S1.

To validate the formation of ISTL1, we used SHAPE, a powerful chemical structure probing technique (42). In this assay, 1M7 modifies the freely available 2′-hydroxyl groups (of sugar moieties) of nucleotides. In general, residues located in loops and bulges are reactive, whereas residues that form stems or engage in high-order interactions are protected from modification. We performed all our structure probing experiments using a 467 nt long RNA harboring TSL2, a structure formed at the 3′-end of exon 7 (16), and the entire intron 7. The secondary structure of *SMN2* intron 7 deduced from the SHAPE analysis showed a substantial overlap with the predicted secondary structure (Figure 6). According to our results, intron 7 folds into several hairpins (TSLs) and internal stems formed by LDIs (ISTLs). Importantly, the results of SHAPE validated the formation of ISTL1 (Figure 6A). For instance, 14 of 16 residues of ISTL1 were largely inaccessible for modification. We detected some degree of modification of 3A and 293G that are located at the terminal position and in the middle of ISTL1, respectively. Of note, examples of SHAPE reactivity for nucleotides located within a stem have been reported (45,46). Validating the formation of TSL2, three base pairs in the middle of the stem of TSL2 were completely protected from modification (Figure 6A). We did observe accessibility of additional residues at the most flexible positions such as loop closing and the base of the stem of TSL2 (Figure 6A).

The probed structure localized ISTL1 adjacent to two internal stems, which we designated as ISTL2 and ISTL3 (Figure 6A). The sequence stretch connecting the 5′ strand of ISTL1 with the 5′ strand of ISTL2 folds into a hairpin structure (TSL3), which partially sequesters the previously reported binding sites of hnRNP A1/A2B1 and TIA1 proteins (Figure 6A). A few nucleotides in the predicted stem of TSL3 were found to be accessible for modification. These include a U residue (25U) in the loop-closing base pair, a G residue (39G) in the wobble base pair and an A residue (18A) in the neighboring base pair (Figure 6A). Residues immediately upstream and downstream of TSL3 are located within internal loops (Figure 6A). It is likely that the formation of these loops is dictated by the topological constraints imposed by ISTL1 and ISTL2. We also identified two regions where the SHAPE results did not corroborate with the *mfold-*predicted structure. These regions constitute Modules 1 and 2 that occupy a major portion of intron 7 (Figure 6). We conclude that the formation of ISTL1 is independent of these modules, as their deletion did not abrogate the inhibitory effect of L14 (Figure 2). Another structural module, Module 3, deletion of which had no consequences on the negative effect of L14, showed substantial agreement between the experimentally derived and the *mfold-*predicted structure (Figure 6). Simultaneous deletions of Modules 1, 2 and 3 retained the inhibitory effect of L14 (Figure 2), suggesting that formation of ISTL1 is feasible even in the context of a short intron 7.

**Figure 7.**
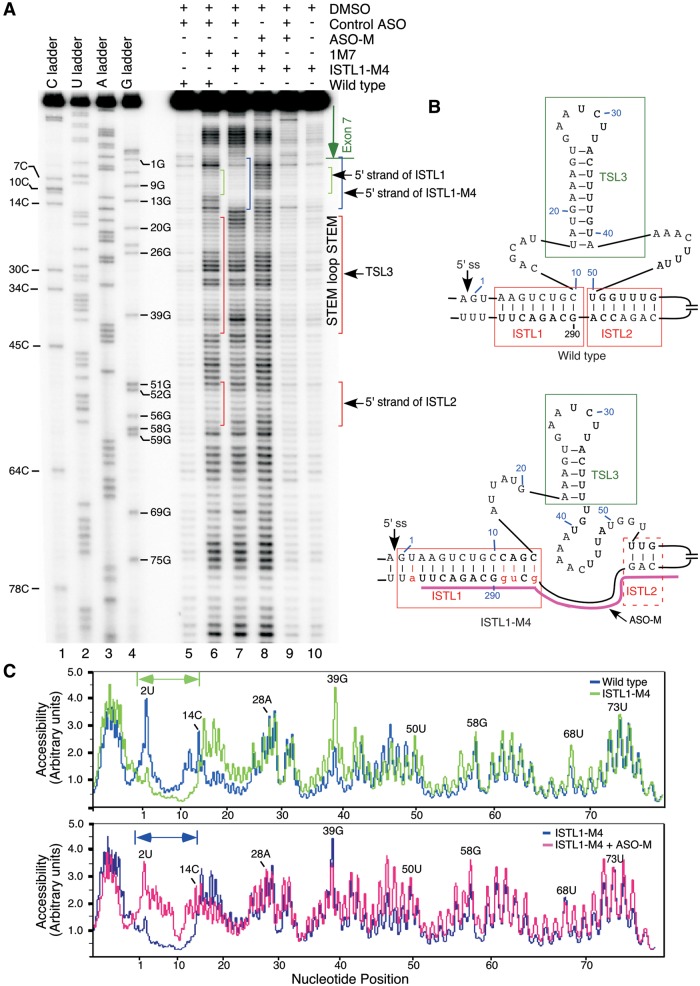
Validation of the engagement of the 5′ strand of ISTL1 in structure formation. (**A**) SHAPE results for the 5′ portion of intron 7 of the wild-type and ISTL1-M4 mutant RNAs generated using Primer#10. Based on the sequencing ladders, positions of residues and locations of structures are marked on the gel. (**B**) Abridged *mfold* predicted structure of *SMN2* intron 7. The probed structure is in agreement with the *mfold* predicted structure. Nucleotide substitutions are shown in small-case letters and highlighted in red. Annealing site of ASO-M is indicated. Abbreviations are the same as in Figure 6. (**C**) Alignment of raw peak profiles for the wild-type and mutant RNAs. Nucleotides that constitute the 5′ strand of ISTL1 in the mutant RNA are marked. Peak profiles were generated using MultiGauge Software version 3.0 (FujiFilm).

To further confirm the formation of ISTL1, we compared the SHAPE profiles of the wild-type and ISTL1-M4 RNAs. As predicted, ISTL1-M4 RNA showed a significantly decreased 1M7 reactivity of the −1A, 1G, 2U, 3A 11C, 12A, 13 G and 14C nucleotides that are located in the extended 5′ strand of ISTL1 (Figure 7A, compare lanes 6 and 7; Figure 7C, upper panel). SHAPE results also indicated that the extended stem in ISTL1-M4 RNA caused perturbations in several local structures. For instance, multiple residues involved in the formation of TSL3 became accessible for modification by 1M7 [Figure 7A and C (upper panel)]. In addition, ISTL2 became destabilized as indicated by the increase in 1M7 modifiability of residues in its 5′ strand, particularly at positions 51 and 56 [Figure 7A and C (upper panel)]. To obtain additional validation that the 5′ and 3′ strands of ISTL1 are protected from modifications owing to base pairing with each other, we performed SHAPE analysis of ISTL1-M4 RNA refolded and probed in the presence of an ASO (ASO-M) that targeted the mutated 3′ strand of ISTL1 (from the 275th to 297th positions of intron 7) (Figure 7B). As expected, the presence of ASO-M increased the reactivity of residues from the last position of exon 7 to the 14th position of intron 7 [Figure 7A (compare lanes 7 and 8) and C (lower panel)]. Interestingly, ASO-M had a much lesser effect on accessibility of C residues at the 10th and 11th positions of intron 7 [Figure 7A and C (lower panel)]. This could be due to engagement of these residues in an alternative interaction when the 3′ strand of ISTL1 is sequestered by ASO-M. In addition, instances in which C residues in flexible regions display lower SHAPE reactivity than other residues with similar apparent local structure have been reported (45). Overall, our results provided the first validated example in which the sequestration of a deep intronic sequence increased the accessibility of the 5′ ss of an exon.

**Figure 8.**
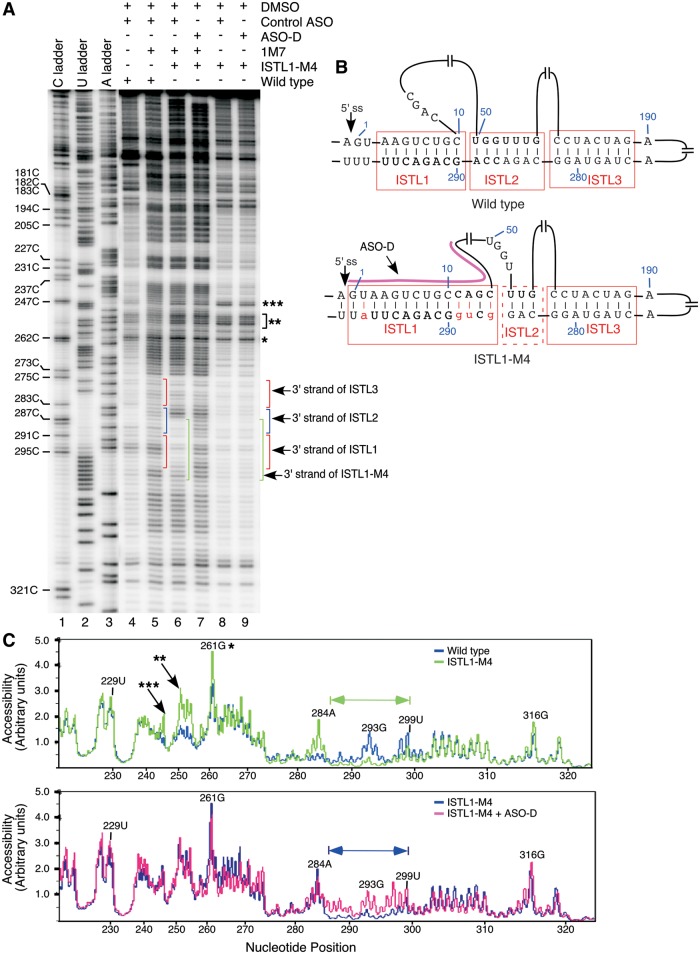
Validation of the engagement of the 3′ strand of ISTL1 in structure formation. (**A**) SHAPE results for the middle portion of intron 7 of the wild-type and ISTL1-M4 mutant RNA generated using Primer#17. Based on the sequencing ladders, positions of residues and locations of structures are marked on the gel. (**B**) Abridged *mfold-*predicted structure of *SMN2* intron 7. The probed structure is in agreement with the *mfold-*predicted structure. Nucleotide substitutions are shown in small-case letters and highlighted in red. Annealing site of ASO-D is indicated. (**C**) Alignment of raw peak profiles of the wild-type and mutant RNAs. Nucleotides that constitute the 3′ strand of ISTL1 in the mutant RNA are marked. Peak profiles were generated using MultiGauge Software version 3.0 (FujiFilm). RTase falloff products are marked by stars.

We also compared the SHAPE profiles of the 3′ strands of ISTL1 in the wild-type and ISTL1-M4 RNAs. Interestingly, we observed a significant falloff of reverse transcriptase (RTase) at positions 292 and 293 in the mutant RNA in both control (DMSO) and 1M7 treated samples that interfered with structure determination (Supplementary Figure S2). We attribute these falloffs to a combination of factors, including the presence of a strong secondary structure (ISTL1) in front of the U-rich tract. To obviate the RTase falloff, we modified our primer extension reaction by adding an ASO (ASO-D), which is predicted to disrupt ISTL1 by sequestering its 5′ strand (Figure 8B). Indeed, the presence of ASO-D fully prevented the falloff of RTase at the 292nd and 293rd positions (Supplementary Figure S2). These results provided additional evidence in support of the formation of ISTL1. With the exception of 293G, residues from the 275th to 297th positions of intron 7 were poorly modifiable by 1M7 owing to their incorporation within ISTLs [Figure 8A (lane 5) and C (upper panel)]. Yet, ISTL1-M4 RNA showed a further decrease in 1M7 reactivity of residues from the 286th to 295th positions of intron 7 [Figure 8A (compare lanes 5 and 6) and C (upper panel)]. Also, reactivity at the 298th and 299th positions decreased in the mutant ISTL1, whereas residues at the 283rd to 285th positions located immediately upstream of the strengthened ISTL1 became moderately to highly modifiable [Figure 8A and C (upper panel)]. As expected, the SHAPE profile generated for the mutant RNA refolded and probed with ASO-D showed an increase in the 1M7 reactivity of nucleotides from the 286th to 299th positions of intron 7, with the exception of 291C [Figure 8A and C (lower panel)]. To summarize, our results of structure probing confirmed the formation of ISTL1.

### Effect of F14 and L14 on stability of ISTL1

**Figure 9.**
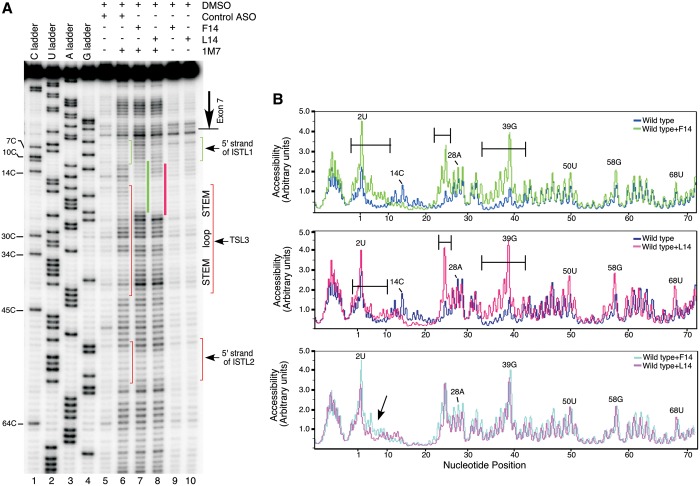
Effect of F14 and L14 on RNA secondary structure of *SMN2* intron 7. (**A**) SHAPE results for the wild-type RNA probed in the presence of F14 and L14. Primer#10 was used to identify 1M7 modification sites. Based on the sequencing ladders, positions of residues and locations of structures are marked on the gel. Annealing positions of F14 and L14 are marked by green and red bars, respectively. (**B**) Alignment of raw peak profiles for the wild-type RNA refolded and probed with F14 or L14. Nucleotides that display higher reactivity toward 1M7 in the presence of F14 and L14 are indicated. The region where nucleotides are more reactive with 1M7 in the presence of F14 than in the presence of L14 is indicated by an arrow.

To capture the effect of F14 and L14 on ISTL1 and other structures of intron 7, we performed SHAPE analysis in which we refolded the wild-type RNA in the presence of F14 or L14 before incubation with 1M7. The SHAPE profiles confirmed the appropriate annealing of F14 and L14 to their respective targets (Figure 9A). As the target sites of both F14 and L14 overlap the 5′ strand of the stem in TSL3, we observed increased 1M7 reactivity of residues from the 33rd to 41st positions of intron 7 in the presence of either F14 or L14 [Figure 9A and B (upper and middle panels)]. In addition, both ASOs caused an increase in modifiability of some of the residues in the region that encompasses the 5′ strand of ISTL2 (Figure 9). Importantly, F14 and L14 caused destabilization of the region upstream of their annealing sites as indicated by an increase in the modifiability of residues in the area extending to the two last positions of exon 7 (Figure 9). Notably, F14 showed a greater destabilizing effect than L14 (Figure 9B, lower panel).

The presence of F14, but not L14, resulted in a slight increase in the modifiability of residues that constitute the 3′ strand of ISTL1 (Supplementary Figure S3). Consistent with the increased 1M7 reactivity of residues between the 50th and 58th positions of intron 7 (Figure 9), we also observed increased modifiability of residues in the complementary 3′ strand of ISTL2 in the presence of both ASOs, particularly at positions 284 and 285 (Supplementary Figure S3). A comparison of SHAPE profiles did not reveal any novel secondary structure whose formation was induced by annealing of either F14 or L14 to their respective targets. However, it is possible that the minor structural differences induced by F14 and L14 may be sufficient to create distinct topological context for the RNA-protein interactions.

### Role of protein factors in ^10^C-mediated LDI

**Figure 10.**
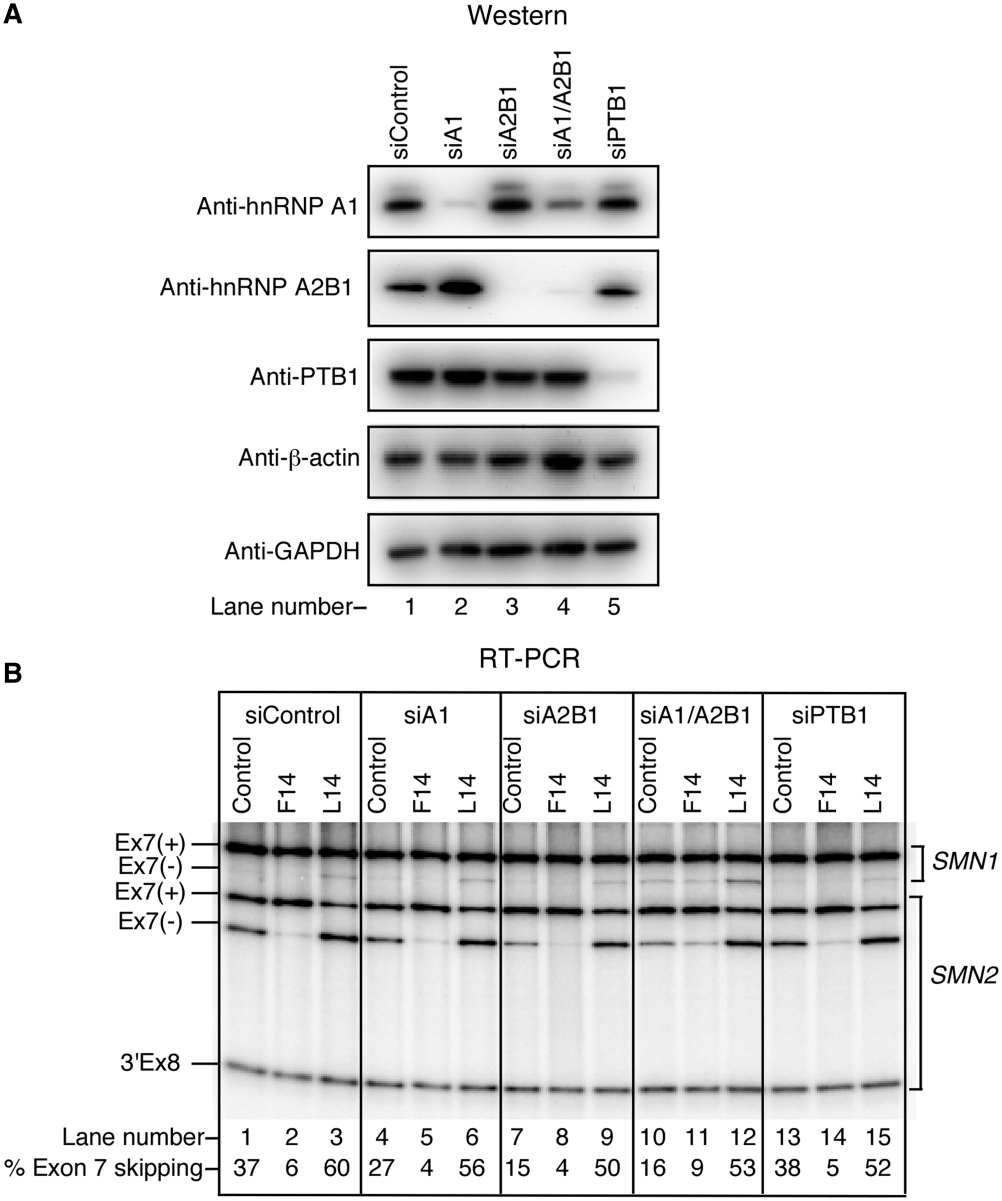
Effect of depletion of hnRNP A1/A2B1 and PTB1 on ^10^C-mediated LDI. (**A**) Western blot results showing the effect of indicated siRNAs on the level of corresponding proteins. (**B**) Splicing pattern of the endogenous *SMN* exon 7 in the presence of the control ASO (10mer), F14 and L14 in HeLa cells treated with different siRNAs. Control ASO was the same as described in Figure 2. Spliced products amplified by RT-PCR were digested with DdeI to distinguish between the transcripts from *SMN1* and *SMN2* pre-mRNA (25). 3′Ex8 represents the cleavage product of DdeI digestion of *SMN2* exon 8. The percentage of *SMN2* exon skipping was calculated as in (28).

The abundantly expressed proteins hnRNP A1/A2B1 and PTB1 have been implicated in skipping of *SMN2* exon 7 (27). Owing to the proximity of ISS-N1 to ISTL1 in the folded RNA, there is a probability that the *in vivo* interaction of hnRNP A1/A2B1 with ISS-N1 is facilitated by ISTL1. It is also possible that hnRNP A1/A2B1 assists folding of or stabilizes ISTL1. Considering the longest U-rich tract of intron 7 is located immediately downstream of the LS-1 sequence (Figure 6), PTB1 may be involved in ISTL1 formation as well. To explore whether hnRNP A1/A2B1 and PTB1 are at all associated with the ^10^C-mediated LDI, we first depleted HeLa cells of these proteins using a siRNA-based approach and then transfected these cells with F14 or L14 followed by determination of splicing pattern of endogenous exon 7 from both *SMN1* and *SMN2*. To distinguish between *SMN1* and *SMN2* transcripts, we took advantage of a DdeI restriction site specific to *SMN2* exon 8 (25). As expected, depletion of either hnRNP A1 or A2B1 produced a noticeable stimulatory effect on *SMN2* exon 7 splicing [Figure 10A and B (lanes 4 and 7)]. In particular, *SMN2* exon 7 inclusion was substantially higher in hnRNP A2B1-depleted compared with hnRNP A1-depleted cells. However, the inhibitory effect of L14 on *SMN2* exon 7 splicing was not abrogated by knockdown of hnRNP A1 and A2B1, individually or together (Figure 10, lanes 6, 9 and 12). Interestingly, depletion of hnRNP A2B1 alone or together with hnRNP A1 produced more than a 3-fold increase in L14-induced skipping of *SMN2* exon 7 compared with only ∼2-fold increase when control siRNA-treated HeLa cells were transfected with L14 (Figure 10B, compare lane 3 with lanes 6, 9 and 12). As hnRNP A1 and A2B1 are involved in several other processes, including but not limited to transcription and microRNA biogenesis (47), we attribute these differences to indirect factors. Similar to hnRNP A1/A2B1, depletion of PTB1 did not eliminate the inhibitory effect of L14 on *SMN2* exon 7 splicing (Figure 10B, lane 15). Therefore, our results clearly ruled out the role of hnRNP A1/A2B1 and PTB1 in the ^10^C-mediated LDI.

### ISTL1 as a potential therapeutic target for splicing correction in SMA

**Figure 11.**
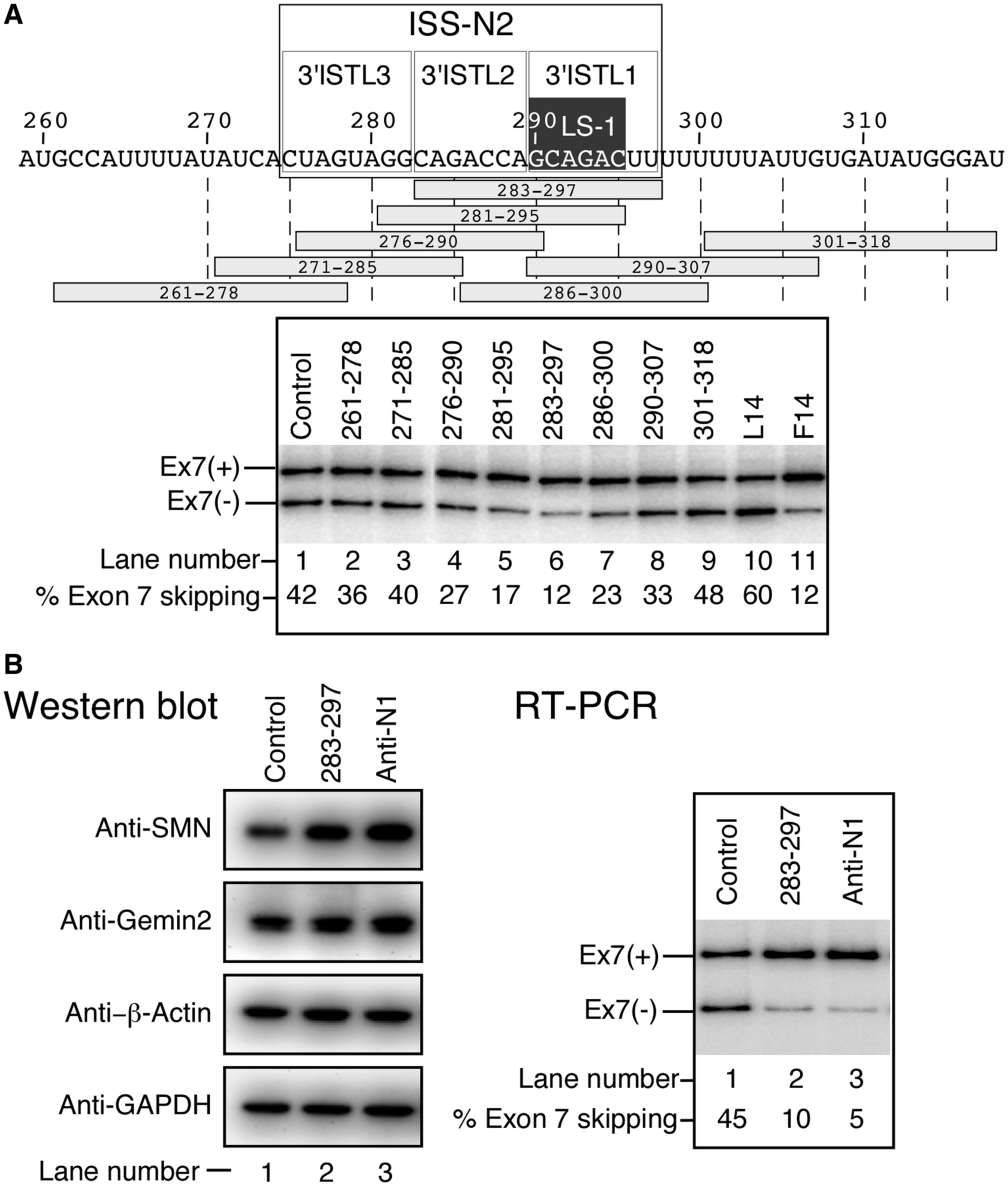
Effect of ASOs on splicing of endogenous *SMN2* exon 7 in SMA patient cells. (**A**) Diagrammatic representation of the region of intron 7 targeted by the indicated ASOs. Numbering of nucleotides starts from the beginning of intron 7. Areas corresponding to the 3′ strands of ISTLs as well as ISS-N2 are indicated. ASOs are shown as horizontal bars. ASO name indicates the first and the last position of their target site in intron 7. SMA patient fibroblasts (GM03813) were transfected with 15 nM of a given ASO and total RNA was harvested at 24 h post transfection. Control ASO was the same as described in Figure 2. Results were analyzed as described in (35). (**B**) Effect of the stimulatory ASOs on levels of cellular proteins in SMA patient cells. GM03813 cells were transfected with 40 nM of a given ASO, and cells were harvested at 48 h post-transfection. Splicing pattern and protein levels were determined as in (35).

The widely used GM03813 cell line (SMA type I fibroblasts) contains only *SMN2* and provides an ideal cell-based disease model to check the efficacy of compounds to modulate *SMN2* exon 7 splicing *in vivo*. To evaluate the impact of ISTL1 in the context of the endogenous gene, we transfected GM03813 cells with ASOs that annealed to overlapping intronic targets encompassing the 3′ strand of ISTL1 and neighboring sequences. To avoid off-target effects, we performed this experiment at a low nanomolar (15 nM) concentration of ASOs. Supporting the inhibitory nature of ISTL1, ASOs that sequestered the 3′ strand of ISTL1 promoted *SMN2* exon 7 inclusion (Figure 11A, lanes 4–8). ASO 283-297, which sequestered the entire 3′ strands of ISTL1 and ISTL2, emerged as the most effective ASO, whose stimulatory effect was comparable with that of F14, which targeted ISS-N1 (Figure 11A, lanes 6 and 11). ASO 276–290, which predominantly targeted the 3′ strands of ISTL2 and ISTL3, also stimulated exon 7 inclusion, albeit to a substantially lower extent (Figure 11A, lane 4). This reduced response may be due in part to partial destabilization of the neighboring ISTL1 and/or TSL3. For the convenience of distinction from our previously reported antisense target ISS-N1 (34), we designate sequences from the 275th to 297th position of intron 7 as ISS-N2 (Figure 11A, upper panel). The inhibitory nature of ISS-N2 is fully supported by the results of antisense microwalk (Figure 11A) and the overlapping deletions (Figures 2 and 3). Similar to ASO 283–297, a 23 nt long ASO that fully sequestered ISS-N2 stimulated exon 7 inclusion (not shown).

We also examined the effect of ASO 283–297 on the levels of SMN in SMA patient cells. As a positive control, we used a 20mer ASO (Anti-N1) that is known to stimulate *SMN2* exon 7 inclusion by binding to ISS-N1 (34). As a negative control, we used a 10mer ASO described previously (40). We transfected GM03813 cells with 40 nM of a given ASO and determined the splicing pattern of *SMN2* exon 7 as well as the protein levels at 48 h post-transfection. As expected, ASO 283–297 effectively stimulated *SMN2* exon 7 inclusion and upregulated SMN protein levels in SMA patient cells (Figure 11B). We also observed a noticeable increase in the level of SMN-interacting protein Gemin2 (Figure 11B, left panel). The stimulatory effect of ASO 283–297 on *SMN2* exon 7 splicing and levels of SMN and Gemin2 was comparable with that of Anti-N1 (Figure 11B). Taken together, these results represent the first example in which an ASO annealing to a deep intronic sequence corrects aberrant splicing and restores high levels of a full-length protein in a cell-based model of a genetic disease.

## DISCUSSION

SMA is the leading genetic cause of infant mortality. The disease has the potential to be corrected through prevention of *SMN2* exon 7 skipping. Our previous discovery of the 15 nt long ISS-N1 has emerged as the most promising target for an ASO-mediated restoration of *SMN2* exon 7 inclusion (34–39). This study began with the aim to uncover how two 14mer ISS-N1-targeting ASOs (F14 and L14) produced opposite effects on alternative splicing of *SMN2* exon 7. We established that the inhibitory effect of L14 is exclusively linked to the unsequestered ^10^C and is contingent on the presence of a downstream intronic sequence separated from ^10^C by hundreds of nucleotides; there was no precedence of any similar finding. To identify the motif associated with the ^10^C-mediated LDI, we adopted an unbiased approach in which we tested the effect of L14 on splicing of *SMN2* minigene mutants harboring large deletions and 20 nt long overlapping deletions within the entire 3′-half of intron 7. Our results delineated a single 19 nt long sequence stretch from the 282nd to 300th positions of intron 7 as the primary site of the LDI associated with ^10^C (Figure 2). Subsequent screening of minigenes with shorter overlapping deletions and with point mutations narrowed the site of the LDI to a 6 nt long motif (LS-1) that spans from the 290th to 295th positions of intron 7 (Figures 3 and 4). LS-1 is located within the 3′ strand of an *mfold*-predicted structure we term ISTL1 (Figure 4A). The 5′ strand of ISTL1 harbors ^10^C, which base pairs with the first residue (290G) of LS-1 (Figure 4A). Another motif identical to LS-1 (GCAGAC) spans the 282nd to 287th positions of intron 7. Interestingly, deletion of LS-1 (Δ290–295) and not the identical upstream sequence (Δ282–287) abrogated the formation of ISTL1 and led to the complete loss of the inhibitory effect associated with L14 (Supplementary Figure S1). These results underscored the critical role of structural context of LS-1 for the realization of the inhibitory effect associated with ^10^C. Consistent with this statement, all intronic deletions that retained the inhibitory effect of L14 maintained the formation of ISTL1.

Compared with ISS-N1 deletion that produced a strong stimulatory effect on *SMN2* exon 7 splicing (34), deletion of LS-1 and intronic sequences immediately upstream of LS-1 produced a moderate but noticeable stimulatory effect on exon 7 splicing [Figure 2B, lanes 7, 10 and 13; Figure 3A, lanes 7, 10, 13 and 16; Figure 3B (lanes 7, 10, 13 and 16) and C (lanes 13 and 16)]. However, owing to potential creation of artificial *cis*-element and/or relocation of the existing cis-elements, results of deletion mutations should be interpreted with caution. For instance, as deletion of LS-1 and sequences immediately upstream of LS-1 still retained ISS-N1 that kept the TIA1 binding site away from the 5′ ss of exon 7, it did not have the similar additive stimulatory effect on *SMN2* exon 7 splicing as was observed in case of ISS-N1 deletion. Nonetheless, our results of deletion mutations combined with the enhanced stimulatory effects of F14/L14 on *SMN2* exon 7 splicing produced sufficient enough lead for the identification of ISTL1 that we determined to be the sole facilitator of a unique LDI associated with ^10^C. Interestingly, Δ262–281 and Δ301–320 mutants showed a noticeable increase in *SMN2* exon 7 skipping (Figure 2B, lanes 4 and 16). This could be due to the loss of stimulatory *cis*-elements in these mutants. It is also possible that these mutants somehow strongly enforce the formation of ISTL1.

**Figure 12.**
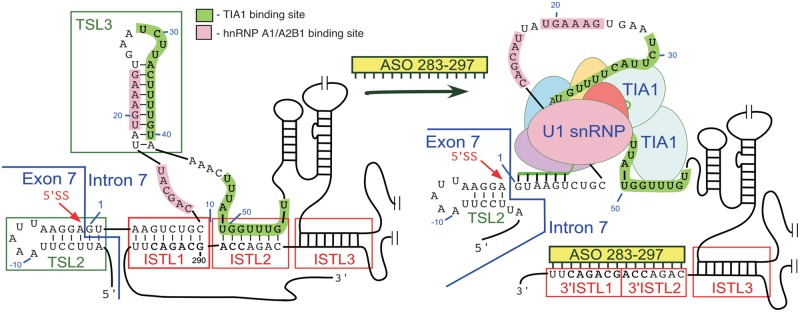
Model of ASO-mediated correction of *SMN2* exon 7 splicing. Only portions of exon 7 and intron 7 are shown (not to scale). Presented structure is based on the SHAPE results (see Figure 6). Structural elements of interest are highlighted. Splicing factors are indicated by colored ovals. Annealing sites of ASO 283–297 (green bar) and U1 snRNA (green line) are shown. Sequestration of the 3′ strands of ISTL1 and ISTL2 by ASO 283–297 releases the 5′ strand of ISTL1 and disrupts TSL3. This structural rearrangement leads to enhanced recruitment of U1 snRNP at the 5′ ss of exon 7 and possibly TIA1.

The strongest evidence supporting the role of the ISTL1 structure in the ^10^C-mediated LDI came from the results of compensatory mutations. For example, 8C/292G and 10G/290C mutations, which altered the composition of ISTL1 but maintained the structure, were able to restore the inhibitory effect of L14 (Figure 5B). These results also precluded the role of a linear motif encompassing the 10th position of intron 7 in ^10^C-mediated LDI. Although competing intronic RNA secondary structures have been predicted to play a positive role in the selection of mutually exclusive exons during splicing of the insect Dscam pre-mRNA (8), our results of compensatory mutations provide the first validated evidence that an intra-intronic RNA secondary structure formed by a LDI can play a negative role in splicing regulation. Previous studies have suggested that the weak 5′ ss of *SMN2* exon 7 is due to the presence of a local secondary structure (TSL2) and the close proximity of the 5′ ss to hnRNP A1/A2B1 binding motifs within ISS-N1 (16,26–28,48). The 5′ strand of ISTL1 sequesters four of the six residues involved in base pairing with U1 snRNA, a component of U1 snRNP, recruitment of which is critical for the definition of the 5′ ss of exon 7 [(16), Figure 12]. Supporting the negative role of ISTL1, mutations that extended the length of ISTL1 led to an increased skipping of *SMN2* exon 7 (Figure 5A). Therefore, our discovery of ISTL1 reveals an additional layer of control of *SMN2* exon 7 splicing, where a deep intronic sequence may affect recruitment of U1 snRNP by a direct interaction with the 5′ ss.

Having determined the functional significance of ISTL1, we next performed SHAPE analysis of a large RNA substrate harboring the entire intron 7 and the upstream exonic sequences encompassing TSL2. We also compared the structure of the wild-type RNA with the ISTL1-M4 mutant that possessed a significantly strengthened ISTL1. The results of structure probing validated the formation of ISTL1. Consistently, the ISTL1-M4 mutant, predicted to greatly extend the length of ISTL1, showed protection of additional residues involved in the extension of the 5′ and 3′ strands of ISTL1 (Figures 7 and 8). The SHAPE profile of ISTL1-M4 confirmed that mutations deep inside intron 7 are able to affect the structure of a region located hundreds of nucleotides away, which is possible only when ISTL1 is formed (Figures 7 and 8). Overall, the pattern of 1M7-modified positions supported that intron 7 folds into a complex structure, including the formation of multiple adjacent helices (Figure 6). Considering residues at the interface of adjacent helices generate co-axial stacking that imposes topological constraints on 3D helix organization (49–51), we predict the existence of complex tertiary interactions involving loop-to-loop contacts. Consistently, SHAPE results indicated the presence of large terminal and internal loops as well as extended single-stranded regions. Interestingly, two of the three reported intronic binding sites of hnRNP A1/A2B1 were localized in the structurally accessible regions of intron 7 (Figure 6). On the other hand, most of the U-rich motifs associated with TIA1 were trapped within stems of TSL3 and ISTL2 (Figure 6). Our findings suggest that the structural context of intron 7 is not conducive for the recruitment of TIA1, a critical positive regulator of *SMN2* exon 7 splicing (28). Of note, TIA1 is the sole splicing factor whose mutation has been recently linked to defective *SMN2* exon 7 splicing in the context of a genetic disease (52).

ISTL1 formation requires looping out of a 279 nt sequence, of which 189 residues are occupied by the independently folding Modules 1 and 2 (Figure 6). A 147 nt long sequence downstream of ISTL1 contains another independently folding module (Module 3) that occupies 89 residues of intron 7 (Figure 6). Simultaneous deletion of Modules 1, 2 and 3 reduced the size of intron 7 by more than half and yet preserved the inhibitory effect of L14 (Figure 2). These results demonstrate that the formation of ISTL1 is independent of most of the local secondary structures within intron 7. However, the relative positioning of ISTL1 with respect to ISTL2 appears to modulate other local structures, particularly TSL3. This is evident in the ISTL1-M4 mutant, in which residues involved in the formation of TSL3 become modifiable by 1M7 (Figure 7). Of note, strengthening of ISTL1 in this mutant did not sequester residues directly engaged in TSL3 formation and yet led to perturbation of TSL3. An increase in length of the ISTL1 helix in the ISTL1-M4 mutant also destabilized ISTL2 (Figures 7 and 8). These results suggest that the ISTL1 and ISTL2 in the wild-type context have stabilizing roles in folding of local structures. Such roles for these structures would be analogous to tertiary interactions that are known to dictate the accuracy of local folding (50).

On comparing the 1M7 modification profiles of RNA refolded and probed in the presence of F14 and L14, we observed a link between destabilization of ISTL1 and a stimulatory effect of the ASOs on *SMN2* exon 7 splicing. Although both F14 and L14 are predicted to generate a 14 bp long helix when bound to their respective targets, the F14 helix also invades ISTL1. Therefore, we found that F14 caused a somewhat greater increase in 1M7 modifiability of residues associated with ISTL1 as compared with L14 (Figure 9). It is possible that the co-axial stacking of residues at the interface of the L14-duplex and ISTL1 brings topological constraint that prevents recruitment of U1 snRNP at the 5′ ss of exon 7. Consistently, substitution at the 10th intronic position has been shown to fully eliminate the inhibitory effect of L14 (40). Although both F14 and L14 disrupt TSL3, the constraint of co-axial stacking at the interface of ISTL1 and L14-duplex are likely to provide a different orientation of the TIA1-binding site located within the disrupted TSL3. Therefore, the inhibitory effect of L14 might be due to the unfavorable conformational changes that prevent recruitment of positive regulatory factors.

Previous reports have implicated hnRNP A1/A2B1 and PTB1 in skipping of *SMN2* exon 7 (27,28,48). Incidentally, these abundantly expressed proteins are also involved in a looping mechanism that brings distantly located intronic motifs in close proximity (2,53). To examine whether the inhibitory effect of L14 is linked to hnRNP A1/A2B1 and/or PTB1, we assessed the effect of L14 on splicing of endogenous *SMN2* exon 7 in HeLa cells depleted of these proteins. Our results did not reveal any significant change in the negative effect of L14, suggesting that these proteins are not involved in L14-induced skipping of *SMN2* exon 7 (Figure 10). Based on our results, it is likely that the ISTL1-assisted sequestration of the 5′ ss is a sufficient enough trigger to promote *SMN2* exon 7 skipping. RNA helicases that bind and/or change the orientation of a RNA helix have been implicated in regulation of alternative splicing in pathological conditions (54,55). Future experiments will determine whether RNA helicases affect *SMN2* exon 7 splicing by targeting ISTL1.

Having determined that ISTL1 imparts a negative effect on *SMN2* exon 7 splicing by sequestration of the 5′ ss of exon 7, we next evaluated the potential of ISTL1 as a target for an ASO-mediated splicing correction in SMA patient cells. Sequestration of the 3′ strand of ISTL1 by an ASO is predicted to make the 5′ ss of *SMN2* exon 7 accessible for base pairing to U1 snRNA, a component of U1 snRNP (Figure 12). In addition, TIA1 is known to enhance the recruitment of U1 snRNP to the 5′ ss of exons (56). Therefore, we hypothesize that the sequestration of the 3′ strand of ISTL2 might also promote U1 snRNP recruitment by releasing TIA1-binding sites located within the helices of ISTL2 and TSL3 (Figure 12). Hence, we expected to see a better stimulatory effect on *SMN2* exon 7 splicing when the 3′ strands of ISTL1 and ISTL2 were sequestered simultaneously. Indeed, a 15 nt long ASO (ASO 283–297) that targeted the 3′ strands of ISTL1 and ISTL2 significantly increased *SMN2* exon 7 inclusion (Figure 11A). Consistent with the stimulatory effect of ASO 283–297 on *SMN2* exon 7 splicing, this ASO also increased the levels of SMN in SMA patient cells (Figure 11B). SMN has high affinity for Gemin2. SMN:Gemin2 interaction has been considered to be one of the critical steps for the assembly of all SMN complexes (57,58). Consistent with the increase in SMN, we found increased levels of Gemin2 in SMA patient cells treated with ASO 283–297. Most importantly, the stimulatory effect of ASO 283–297 was found to be comparable with Anti-N1, a 20mer ASO that targets ISS-N1.

Although we have used only a 15mer ASO to demonstrate the therapeutic potential of a deep intronic target, our results of deletion mutations support a larger inhibitory region that we term ISS-N2, which encompasses the 3′ strands of ISTL1, ISTL2 and ISTL3 (Figure 11A). Therefore, different oligonucleotide chemistries, particularly those that work better for longer ASOs, may be more useful when targeting the entire ISS-N2. Presently, SMA has no cure. An ISS-N1-targeting ASO with phosphorothioate backbone and 2-O-methoxyethyl (MOE) modification, a proprietary of ISIS Pharmaceuticals, is currently undergoing the 2nd phase of clinical trial (Clinicaltrials.gov ID NCT01839656). For SMA therapy, our finding of ISS-N2 comes at a time when lack of additional targets has prevented clinical trials of ASOs with other promising chemistries that have shown encouraging results in recent pre-clinical and clinical trials (39,59). There is a substantial difference in sequence composition between ISS-N1 and ISS-N2. In view of the fact that sequence composition alone can affect pharmacokinetics and pharmacodynamics of an ASO, availability of ISS-N2 as a novel target has additional significance for developing an entirely new class of therapeutic ASOs for the treatment of SMA.

The coding sequence of mammalian *SMN* is mostly conserved. However, there is a noticeable divergence among intronic sequences. For example, mouse *Smn* intron 7 is 1640 nt long, whereas human *SMN* intron 7 is only 444 nt long. In addition to the absence of ISS-N1, mouse *Smn* intron 7 lacks ^10^C (34). Consistently, the *mfold*-predicted structure of mouse *Smn* intron 7 did not reveal a structure analogous to ISTL1/ISTL2/ISTL3 (not shown). Our results combined with the prior reports clearly suggest an evolutionarily distinct intra–intronic regulatory network that fine tunes and modulates with utmost precision the splicing of human *SMN* exon 7 [Figures 6 and 12, Supplementary Figure S4, (32)]. Currently, the literature is replete with studies that focus on linear *cis*-elements, particularly in the beginning of introns (60,61). Our findings suggest that the information content of a linear *cis*-element at the beginning of intron can be tightly controlled by a deep intronic sequence. About 50% of all genetic disorders are caused by mutations that alter pre-mRNA splicing (62). Intronic sequences occupy a major portion (∼26%) of human genome and represent ∼94% of pre-mRNA (63). There is growing appreciation that a significant portion of the transcriptome’s regulatory information is trapped in secondary and high-order RNA structures (64). Our discovery of ISTL1 provides the proof of principle how the structural information locked in the deep intronic sequence could impact the context of the 5′ ss, which is often populated by a multitude of linear *cis*-elements. Our results also demonstrate that an ASO-mediated sequestration of a deep intronic sequence could remodel the 5′ ss by disrupting a LDI associated with an RNA structure. While these findings undoubtedly advance our understanding of splicing regulation in SMA, they also signify the enormous potential for uncovering the structure-associated regulatory network of general splicing.

## SUPPLEMENTARY DATA

Supplementary Data are available at NAR Online.

## FUNDING

National Institutes of Health (NIH) [NS055925, NS072259 and NS080294] and Salsbury Endowment (Iowa State University, Ames, IA, USA) (to R.N.S.). Funding for open access charge: NIH grants and Salsbury Endowment.

*Conflict of interest statement.* ISS-N1 target (US patent # 7,838,657) was discovered in the Singh lab at UMASS Medical School (Worcester, MA, USA). Inventors, including R.N.S., N.N.S. and UMASS Medical School, are currently benefiting from licensing of ISS-N1 target to ISIS Pharmaceuticals. Iowa State University has filed invention disclosure with US patent office on ISS-N2 as a novel therapeutic target for developing SMA drug. Therefore, inventors including R.N.S., N.N.S. and Iowa State University could potentially benefit from any future commercial exploitation of ISS-N2.

## Supplementary Material

Supplementary Data
