# Supplementary material for: An intronic structure enabled by a long-distance interaction serves as a novel target for splicing correction in spinal muscular atrophy
Source: Nucleic Acids Res. 2013 Jul 15;41(17):8144–65. doi: 10.1093/nar/gkt609 (PMC3783185; doi:10.1093/nar/gkt609)
Supplement: Supplementary Data [file supp_41_17_8144__index.html]

An intronic structure enabled by a long-distance interaction serves as a novel target for splicing correction in spinal muscular atrophy — An intronic structure enabled by a long-distance interaction serves as a novel target for splicing correction in spinal muscular atrophy — Supplementary Data 

# An intronic structure enabled by a long-distance interaction serves as a novel target for splicing correction in spinal muscular atrophy

## 

files

**Files in this Data Supplement:**

- Supplementary Data - pdf file
