# Supplementary material for: An intronic structure enabled by a long-distance interaction serves as a novel target for splicing correction in spinal muscular atrophy
Source: Nucleic Acids Res. 2013 Jul 15;41(17):8144–65. doi: 10.1093/nar/gkt609 (PMC3783185; doi:10.1093/nar/gkt609)
Supplement: Supplementary Data [file supp_gkt609_nar-01372-a-2013-File014.pdf]

SUPPLEMENTARY FIGURES

Figure S1

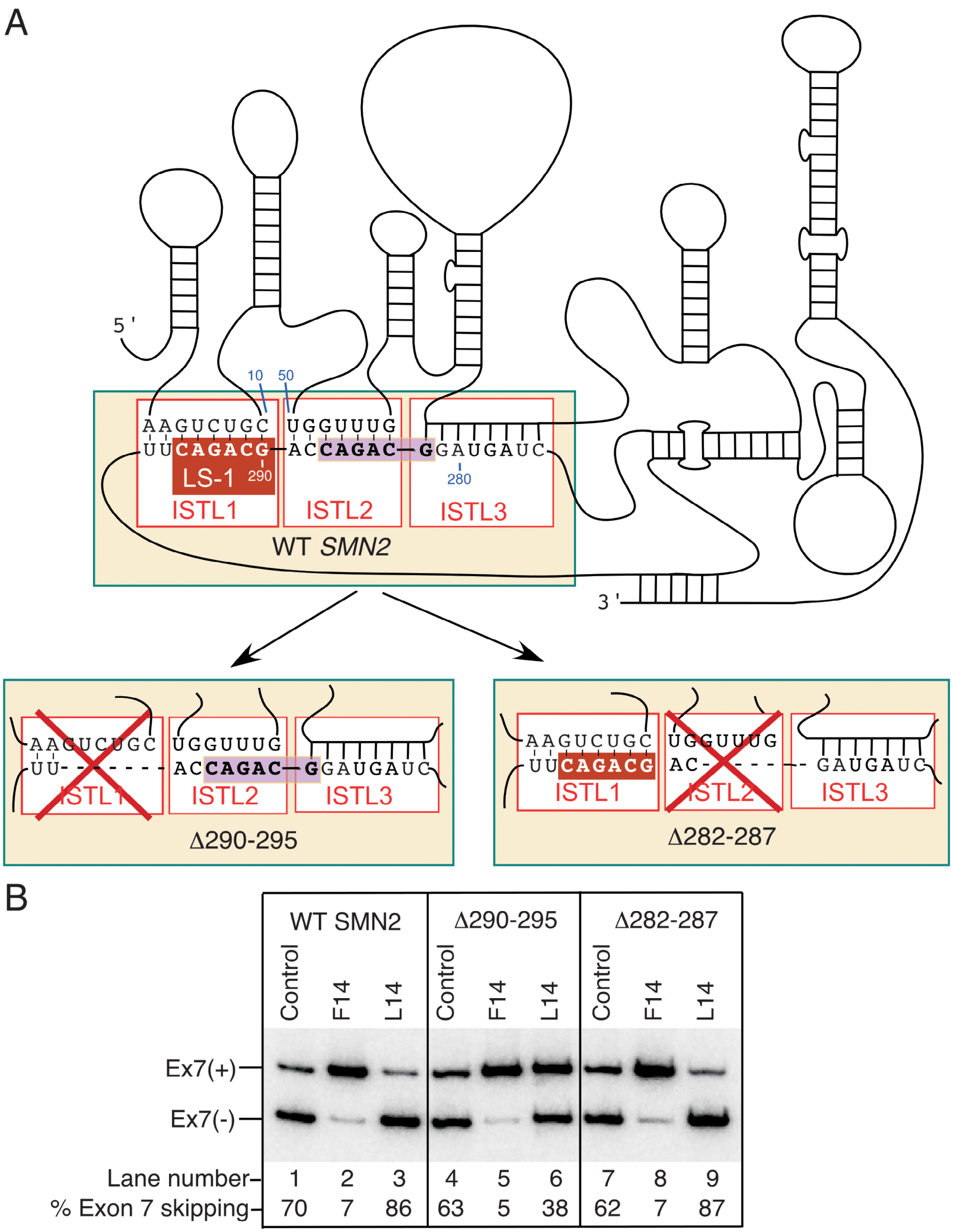

Figure S2

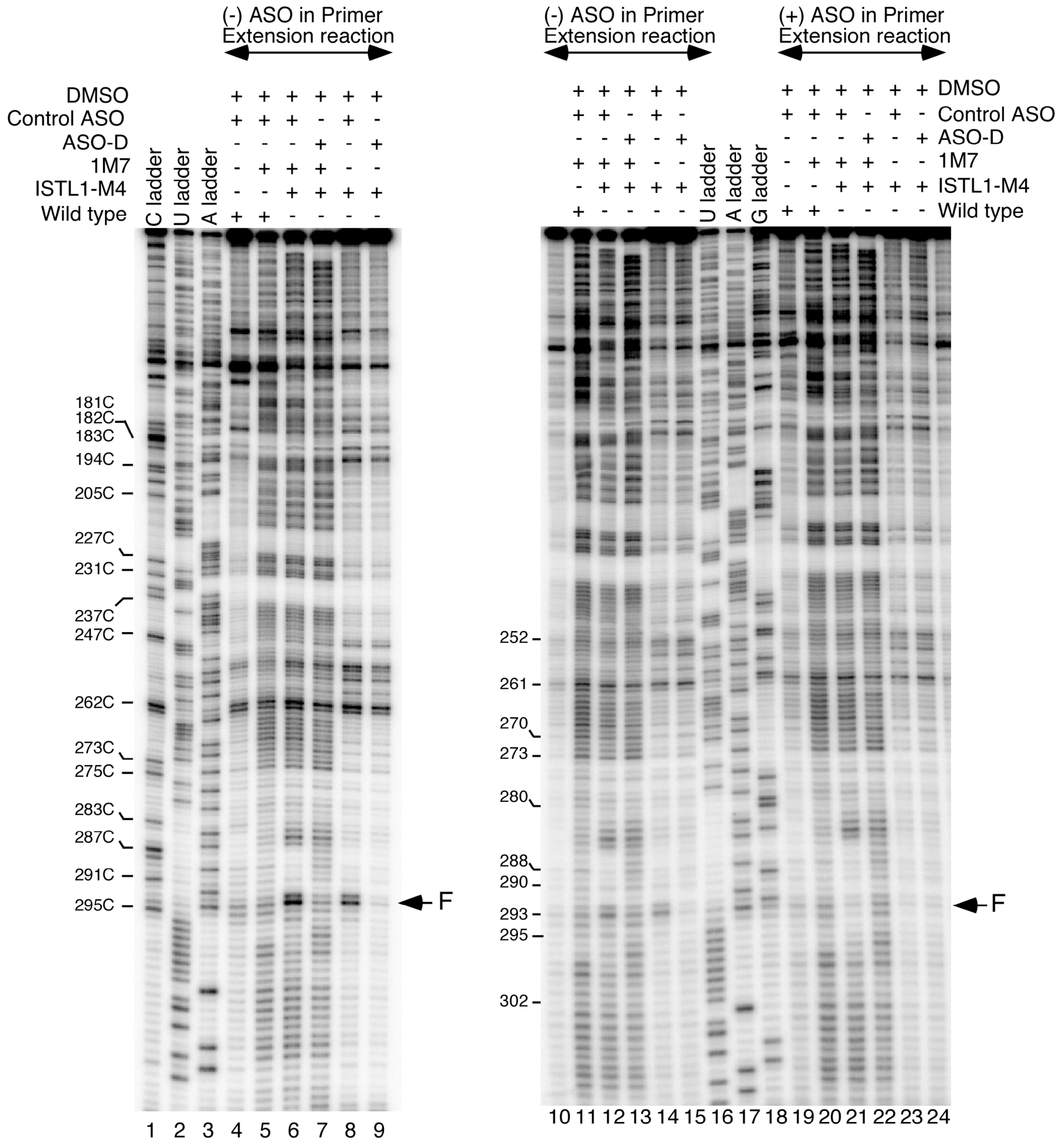
(-) ASO in Primer Extension reaction
DMSO
Control ASO
ASO-D
1M7
ISTL1-M4
Wild type
C ladder
U ladder
A ladder
181C
182C
183C
194C
205C
227C
231C
237C
247C
262C
273C
275C
283C
287C
291C
295C
F
1 2 3 4 5 6 7 8 9
(-) ASO in Primer Extension reaction
(+) ASO in Primer Extension reaction
U ladder
A ladder
G ladder
252
261
270
273
280
288
290
293
295
302
F
10 11 12 13 14 15 16 17 18 19 20 21 22 23 24

Figure S3A

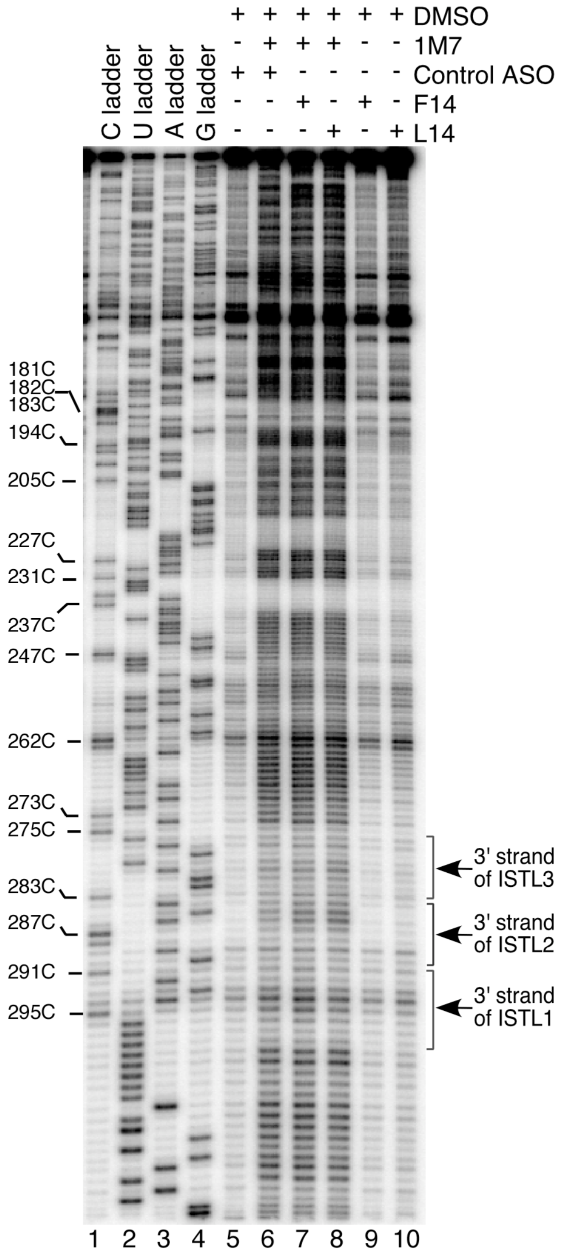
+ + + + + + DMSO
- + + + - - 1M7
+ + - - - - Control ASO
- - + - + - F14
- - - + - + L14
C ladder
U ladder
A ladder
G ladder
181C
182C
183C
194C
205C
227C
231C
237C
247C
262C
273C
275C
283C
287C
291C
295C
3' strand of ISTL3
3' strand of ISTL2
3' strand of ISTL1
1 2 3 4 5 6 7 8 9 10

Figure S3B

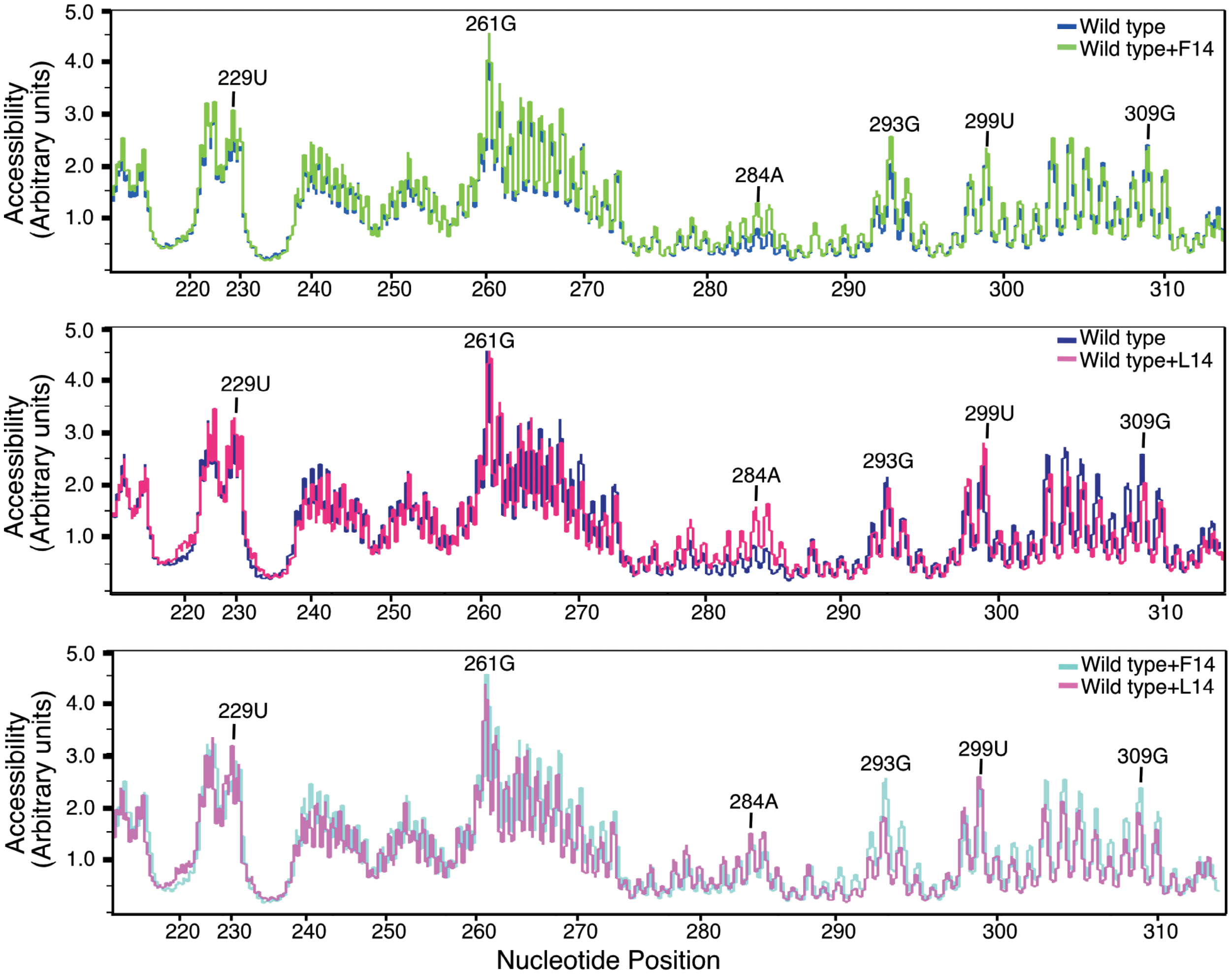

5.0
4.0
3.0
2.0
1.0
Accessibility (Arbitrary units)
Wild type
Wild type+F14
261G
229U
293G
299U
309G
284A
220
230
240
250
260
270
280
290
300
310
Wild type
Wild type+L14
Wild type+F14
Wild type+L14
Nucleotide Position

Figure S4

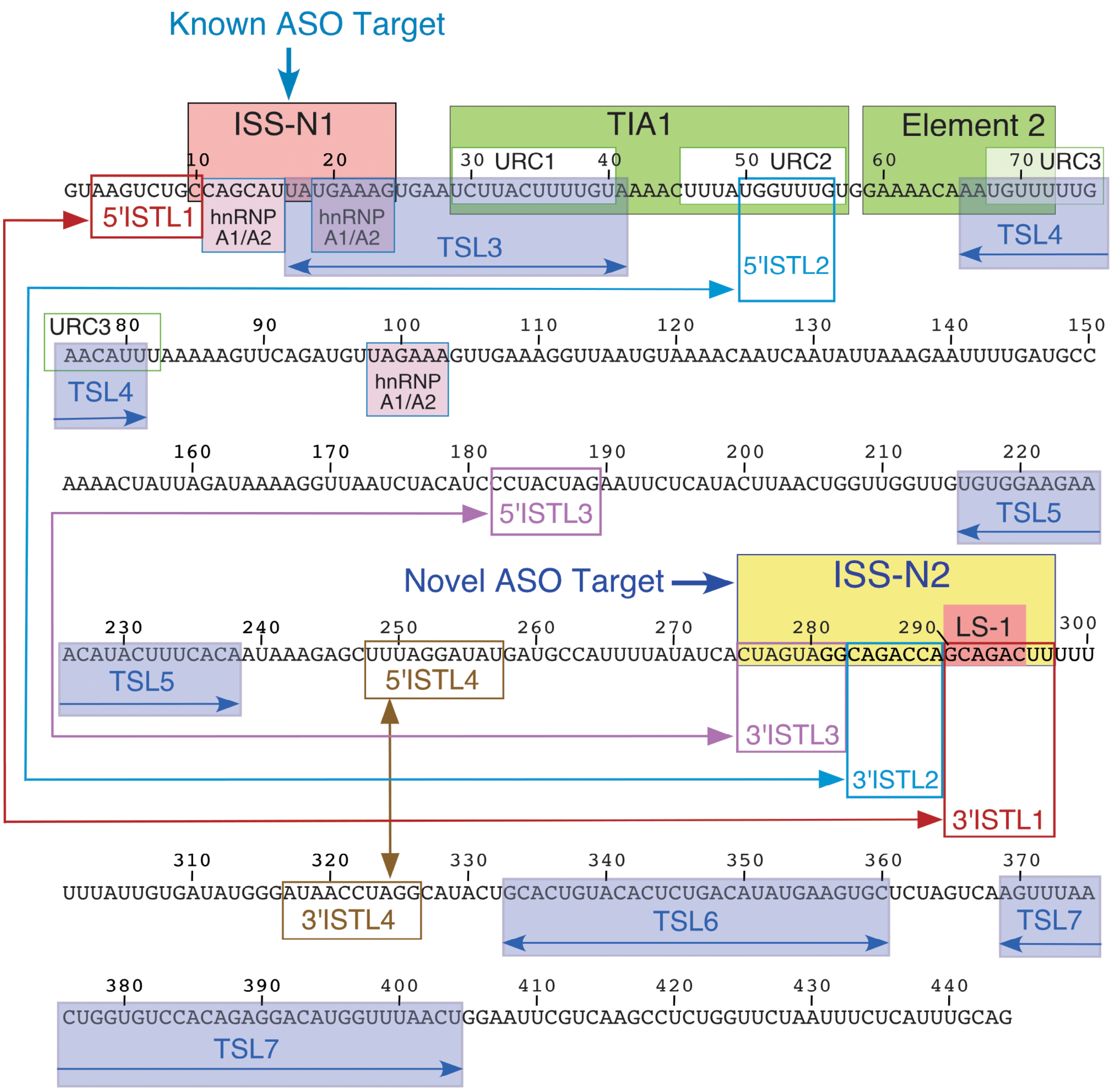

Known ASO Target
ISS-N1
TIA1
Element 2
URC1
URC2
URC3
GUAAGUCUGCCAGCAUUAUGAAAGUGAAUCUUACUUUUGUAAAACUUUAUGGUUUGUGGAAAACAAAUGUUUUUG
5'ISTL1
hnRNP A1/A2
hnRNP A1/A2
TSL3
TSL4
5'ISTL2
AACAUUUAAAAAGUUCAGAUGUUAGAAAGUUGAAAGGUUAAUGUAAAACAAUCAAUAUUAAAGAAUUUUGAUGCC
TSL4
hnRNP A1/A2
AAAACUAUUAGAUAAAAGGUUAAUCUACAUCCUACUAGAAUUCUCAUACUUAACUGGUUGGUUGUGUGGAAGAA
5'ISTL3
TSL5
Novel ASO Target
ISS-N2
LS-1
ACAUACUUUCACAAUAAAGAGCUUUAGGAUAUGAUGCCAUUUUAUAUCACUAGUAGGCAGACCAGCAGACUUUUU
TSL5
5'ISTL4
3'ISTL3
3'ISTL2
3'ISTL1
UUUAUUGUGAUAUGGGAUAACCUAGGCAUACUGCACUGUACACUCUGACAUAUGAAGUGCUCUAGUCAAGUUUAA
3'ISTL4
TSL6
TSL7
CUGGUGUCCACAGAGGACAUGGUUUAACUGGAAUUCGUCAAGCCUCUGGUUCUAAUUUCUCAUUUGCAG
TSL7

SUPPLEMENTARY FIGURE LEGENDS

Figure S1. Effect of GCAGAC motif deletion on the ability of L14 to promote exon 7 skipping. (**A**) Diagrammatic representation of intron 7 structure. Sequence and structural contexts of GCAGAC motifs are highlighted. Numbering of nucleotides starts from the beginning of intron 7. (**B**) In vivo splicing pattern of the wild type *SMN2* minigene and the deletion mutants shown in panel (**A**) in the presence of the indicated ASOs. Deletion of GCAGAC motif in the region from position 290 to 295 but not from 282 to 287 abrogated the negative effect of L14 on exon 7 splicing. Exon 7-included (+) and exon 7-skipped (-) spliced products are indicated. Control represents transfection with 10-mer ASO (Supplementary Table S2). Results were analyzed as described Figure 2B. Abbreviation Ex7 stands for exon 7.

Figure S2. Strengthening of ISTL1 structure in the mutant is corroborated by falloff products in primer extension reactions. The wild type and ISTL1-M4 mutant RNAs were subjected to 1M7 modification. Modification sites were identified by primer extension using primer#17 (left panel) or primer#315 (right panel). Extension reactions with primer#17 and primer #315 were performed on RNA substrates generated and subjected to 1M7 modification at different times. Primer extension products were separated on denaturing 6% polyacrylamide gels. Presence and absence of ASO-D in primer extension reactions are indicated by (+) and (-), respectively. “Falloff” bands corresponding to positions 292 and 293 in both 1M7 treated and untreated mutant RNA are indicated by arrows (lanes 6 and 8 for primer#17 and lanes 12 and 14 for primer#315). When ASO-D was added to primer extension reaction, these bands disappeared in ISTL1-M4 samples (lanes 21 to 23 for primer#315; also see Figure 8, lanes 6 and 8 for primer#17). Therefore, we attribute 292 and 293 falloff products to a strong RNA structure.

Figure S3. Effect of F14 and L14 on RNA secondary structure of the middle portion of intron 7 probed by SHAPE. (**A**) SHAPE results for the wild type intron 7 in the presence of F14 and L14. RNA substrate refolded in the presence of the control ASO, F14 or L14 was subjected to 1M7 modification. Modification sites were then identified by primer extension using primer#17. Primer extension products were separated on denaturing 6% polyacrylamide gels. Based on the sequencing ladders, positions of residues and locations of structures are marked on the gel. (**B**)

Alignment of raw peak profiles for the wild type RNA refolded and probed with F14 versus with L14.

Figure S4. Relative positioning of the structural and splicing cis-elements on the linear structure of *SMN2* intron 7. Entire *SMN2* intron 7 sequence is shown. Numbering starts from the first position of intron 7. Double arrow within a box represents a TSL, whereas, a double arrow outside of the box in specific color shows the 5′ and 3′ strands of a particular ISTL. ISS-N2, a novel therapeutic target, is comprised of the 3′ strands of ISTL1, ISTL2 and ISTL3. Descriptions of abbreviations are given in the main body of the text as well as in the Supplementary Table S1.

Supplementary Tables

Table S1. Abbreviations used in this study

Abbreviation	Full Name	Applicable Figure(s)
1M7	1-methyl-7-nitroisatoic anhydride	NA
^{10}C	Cytosine residue at the10th position of *SMN* intron 7	NA
A100G	An adenosine to guanosine substitution at the 100th position of *SMN2* intron 7	1
ASO	Antisense oligonucleotide	NA
C6U	A cytosine to uridine substitution at the 6th position of *SMN2* exon 7	1
Ex	Exon	NA
Exinct	Extended inhibitory context	1
GC-rich	Sequence from the 7th to 14th positions of *SMN* intron 7	1
hnRNP	Hetero-nuclear ribonucleoprotein	1,S4
ISS-N1	Intronic splicing silencer N1	1, 2,4,S4
ISS-N2	Intronic splicing silencer N2	1,11,S4
ISTL1	Internal stem through LDI 1	1,4-9,11,12,S4
ISTL1-M4	Four substitutions that strengthen ISTL1	5,7,8,S2
ISTL2	Internal stem through LDI 2	1,6,11,12,S4
ISTL3	Internal stem through LDI 3	1,6,11,12,S4
LDI	Long distance interaction	1
LS-1	LDI Site 1	1,4-6,11,S4
nt	Nucleotide	NA
RTase	Reverse transcriptase	
SHAPE	Selective 2′-Hydroxyl Acylation analyzed by Primer Extension	NA
SMA	Spinal muscular atrophy	NA
SMN (Italics)	Survival motor neuron gene or transcript	NA
SMN	Survival motor neuron protein	NA
TIA1	T-cell intracellular antigen-1	NA
TSL2	Terminal stem-loop 2	6,12
TSL3	Terminal stem-loop 3	6,S4
TSL4	Terminal stem-loop 4	6,S4
TSL5	Terminal stem-loop 5	6,S4
TSL6	Terminal stem-loop 6	6,S4
TSL7	Terminal stem-loop 7	6,S4
U1 snRNP	U1 small nuclear ribonucleoprotein	12
URC1	U-rich cluster 1	1,S4
URC2	U-rich cluster 2	1,S4
URC3	U-rich cluster 3	1,S4

Table S2. Sequences of antisense oligonucleotides

No.	Name	Sequence (5′ to 3′)
1	Anti-N1#	A*mU*mU*mC*mA*mC*mU*mU*mU*mC*mA*mU*mA*mA*mU*mG*mC*mU*mG*mG
2	F14#	mU*mU*mU*mC*mA*mU*mA*mA*mU*mG*mC*mU*mG*mG
3	L14#	mC*mU*mU*mU*mC*mA*mU*mA*mA*mU*mG*mC*mU*mG
4	Control (10-mer) #	mU*mU*mG*mC*mC*mU*mU*mU*mC*mU
5	ASO 261-278#	mC*mU*mA*mG*mU*mG*mA*mU*mA*mU*mA*mA*mA*mA*mU*mG*mG*mC
6	ASO 271-285#	mC*mU*mG*mC*mC*mU*mA*mC*mU*mA*mG*mU*mG*mA*mU
7	ASO 276-290#	mC*mU*mG*mG*mU*mC*mU*mG*mC*mC*mU*mA*mC*mU*mA
8	ASO 281-295#	mG*mU*mC*mU*mG*mC*mU*mG*mG*mU*mC*mU*mG*mC*mC
9	ASO 283-297#	mA*mA*mG*mU*mC*mU*mG*mC*mU*mG*mG*mU*mC*mU*mG
10	ASO 286-300#	mA*mA*mA*mA*mA*mG*mU*mC*mU*mG*mC*mU*mG*mG*mU
11	ASO 290-307#	mC*mA*mA*mU*mA*mA*mA*mA*mA*mA*mA*mA*mG*mU*mC*mU*mG*mC
12	ASO 301-318#	mA*mU*mC*mC*mC*mA*mU*mA*mU*mC*mA*mC*mA*mA*mU*mA*mA*mA
13	ASO-M#	mA*mA*mG*mU*mC*mU*mG*mC*mC*mA*mG*mC*mC*mU*mG*mC*mC*mU*mA*mC*mU*mA*mG
14	ASO-D¶	ATTCACTTTCATAATGCTGGCAGACTTAC

# These are RNA ASOs synthesized by Dharmacon/ThermoScientific Molecular Biology. Letter m represents O-methyl modification at the 2nd position of a sugar residue and a star (*) represents phosphorothioate modification of the backbone.

¶DNA oligonucleotide

Table S3. Sequences of primers used for RT-PCR

No.	Name	Annealing position	Sequence (5′ to 3′)
1	N-24	*SMN* Exon 6	CCAGATTCTCTTGATGATGCTGATGCTTTGGG
2	P1	Vector-specific	CGACTCACTATAGGCTAGCC
3	P2	*SMN* Exon 8	GCATGCAAGCTTCCTTTTTCTTTCCCAACAC
4	P25	*SMN* Exon 8	CTCGAAGCGGCCGCAGCTCATAAAATTACCA
5	P31	*SMN* Exon 6	CATGAGTGGCTATCATACTG

Table S4. Sequences of primers used for RTase extensions in SHAPE structure probing

No.	Name	Annealing position within *SMN2* intron 7	Sequence (5′ to 3′)
1	Primer #51	51-81	AATGTTCAAAAACATTTGTTTTCCACAAACC
2	Primer#10	88-112	CCTTTCAACTTTCTAACATCTGAAC
3	Primer#107	107-138	CTTTAATATTGATTGTTTTACATTAACCTTTC
4	Primer#181	181-206	AGTTAAGTATGAGAATTCTAGTAGGG
5	Primer#211	211-237	GTGAAAGTATGTTTCTTCCACACAACC
6	Primer#252	252-281	CTACTAGTGATATAAAATGGCATCATATCC
7	Primer#315	315-340	TACAGTGCAGTATGCCTAGGTTATCC
8	Primer#17	338-362	GAGCACTTCATATGTCAGAGTGTAC
9	Primer#3L	411-436	GAGAAATTAGAACCAGAGGCTTGACG
10	Primer#414	414-442	GCAAATGAGAAATTAGAACCAGAGGCTTG
